# Persistent microglial activation and synaptic loss with behavioral abnormalities in mouse offspring exposed to CASPR2-antibodies in utero

**DOI:** 10.1007/s00401-017-1751-5

**Published:** 2017-07-28

**Authors:** Ester Coutinho, David A. Menassa, Leslie Jacobson, Steven J. West, Joana Domingos, Teresa C. Moloney, Bethan Lang, Paul J. Harrison, David L. H. Bennett, David Bannerman, Angela Vincent

**Affiliations:** 1 Nuffield Department of Clinical Neurosciences, University of Oxford, Oxford, UK; 2 Department of Psychiatry, University of Oxford, Oxford, UK; 3 Oxford Health NHS Foundation Trust, Oxford, UK; 4 Department of Experimental Psychology, University of Oxford, Oxford, UK; 5 Nuffield Department of Clinical Neurosciences, John Radcliffe Hospital, Oxford, OX3 9DU UK

**Keywords:** CASPR2, Maternal antibodies, Neurodevelopmental disorders, Autism, Intellectual development, Maternal-to-fetal mouse model

## Abstract

Gestational transfer of maternal antibodies against fetal neuronal proteins may be relevant to some neurodevelopmental disorders, but until recently there were no proteins identified. We recently reported a fivefold increase in CASPR2-antibodies in mid-gestation sera from mothers of children with intellectual and motor disabilities. Here, we exposed mice in utero to purified IgG from patients with CASPR2-antibodies (CASPR2-IgGs) or from healthy controls (HC-IgGs). CASPR2-IgG but not HC-IgG bound to fetal brain parenchyma, from which CASPR2-antibodies could be eluted. CASPR2-IgG exposed neonates achieved milestones similarly to HC-IgG exposed controls but, when adult, the CASPR2-IgG exposed progeny showed marked social interaction deficits, abnormally located glutamatergic neurons in layers V–VI of the somatosensory cortex, a 16% increase in activated microglia, and a 15–52% decrease in glutamatergic synapses in layers of the prefrontal and somatosensory cortices. Thus, in utero exposure to CASPR2-antibodies led to permanent behavioral, cellular, and synaptic abnormalities. These findings support a pathogenic role for maternal antibodies in human neurodevelopmental conditions, and CASPR2 as a potential target.

**Electronic supplementary material:**

The online version of this article (doi:10.1007/s00401-017-1751-5) contains supplementary material, which is available to authorized users.

## Introduction

In the later part of the 19th century, Paul Ehrlich showed, by a series of elegant experiments on rodents, that immunity is transferred from the mother to her fetus [31]. In mammals, this task is undertaken by the neonatal Fc receptor (nFcR) that binds to maternal Immunoglobulin G (IgG) and transposes it into the fetal circulation [28]. This maternally acquired IgG is thought to provide the newborn with short-term adaptive immunity, until the immune system has fully developed. However, both protective and potentially pathogenic antibodies (i.e., antibodies that could target fetal/neonatal proteins) are transferred through this mechanism. This fact is now well established, through animal and human data, in various neonatal autoimmune diseases [21].

A similar mechanism was proposed in autism and other neurodevelopmental disorders [36], and for review, see [10]. The hypothesis is that the passage of a maternal antibody targeting central nervous system (CNS) proteins across the placenta and through a partly permissive fetal blood–brain barrier results in permanent abnormalities in the developing brain. A subgroup of mothers of children with autism spectrum disorders was shown to have antibodies targeting fetal brain proteins important in neurodevelopment in a previous study [4], but the identified targets were intracellular proteins, and are unlikely to be a primary cause.

By contrast, CASPR2 (contactin-associated protein-2) is an appropriate neuronal-surface target for maternal antibodies that could alter neurodevelopment. CASPR2 is clearly important in neuronal migration, as highlighted by the occurrence of cortical dysplasia in patients with an autism spectrum disorder and epilepsy due to *CNTNAP2* (the gene encoding for CASPR2) homozygous mutations [33]. Mutations have also been identified in other neurodevelopmental disorders associated with psychosis, learning disability, or speech impairment [26]. Furthermore, CASPR2-antibodies are potentially pathogenic in adult patients with several neurological disorders, such as neuromyotonia, limbic encephalitis, or Morvan’s syndrome [13].

Recently, in a study of coded gestational samples, we found CASPR2-antibodies in 8 (4.4%) of 181 mothers of children with a diagnosis of mental retardation or other disorders of psychological development compared with 3 (0.9%) of 347 control mothers (*P* = 0.01) [8]. Others [5] have also reported CASPR2-antibodies in a high percentage (37%) of selected sera (brain-reactive) in a subgroup of mothers of autistic children compared with 8–12% in the control groups, and have explored the consequences of in utero exposure to a monoclonal CASPR2-reactive antibody derived from one mother of a child with autism in a mouse model.

To explore the long-term sequelae of in utero exposure to these antibodies, we used our established mouse maternal-to-fetal transfer model [9, 15] using human IgG preparations from two patients with CASPR2-antibody encephalitis and from three healthy individuals. We systematically studied the maternal-to-fetal transfer of the antibodies, their binding to embryonic mouse brain, and the behavioral and neuropathological consequences.

## Materials and methods

### Human samples and IgG purification

Plasma exchange samples (obtained during periods of disease exacerbation) of two male patients with CASPR2-positive encephalitis (CASPR2-IgG) and sera kindly donated from three healthy age- and sex-matched individuals (HC-IgG) were used in this animal model. Written informed consent was obtained from both patients and healthy controls. Human IgG was purified through the ammonium sulphate precipitation method to avoid possible damage to the Fc domains during elution from Protein-G columns [17]. The preparations were extensively dialyzed against Hartmann’s solution, concentrated by dialysis against polyethylene glycol, and filter-sterilized. IgG concentration was determined using a commercially available human IgG radial immunodiffusion kit (The Binding Site, Birmingham, UK). Antibody levels were determined using a live cell-based assay expressing EGFP-tagged human CASPR2, as previously reported [13]. A summary of the details of the patients, plasma antibody titers, number of injected dams, and injected material for each experiment are provided in Online Resource 1 and 2.

**Fig. 1:**
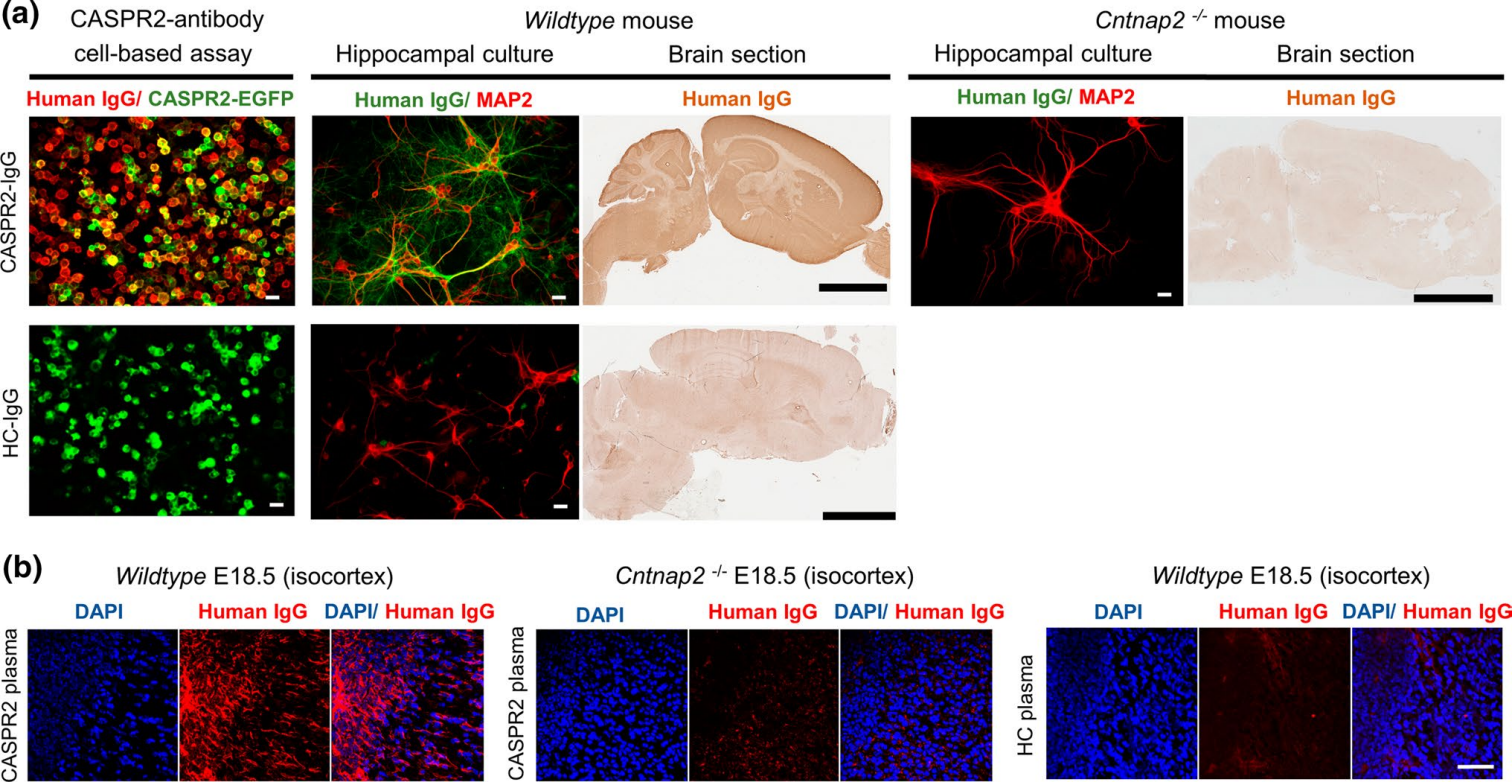
Specificity of CASPR2-IgG. **a** CASPR2-IgG detected by anti-human IgG (*red*) binds to HEK cells expressing EGFP-tagged human CASPR2 (*green*), live hippocampal neurons (neuronal marker: MAP2, *red*; CASPR2-IgG, *green*), and tissue sections from wildtype but not from *Cntnap2* knockout mouse brains. HC-IgG shows no binding to CASPR2-expressing HEK cells, wild-type hippocampal neurons, or wild-type brain sections. **b** CASPR2 plasma IgG (*red*) bind to tissue sections from E18.5 wildtype but not from *Cntnap2* knockout mouse brains. HC plasma IgG (*red*) shows no binding to sections from E18.5 wild-type mouse brain. Representative images using material from CASPR2 patient 1 and HC 1. *Scale bar* (*white*): 25 µm; scale bar (*black*): 3 mm

Specificity of binding to CASPR2 was assessed for all samples used. The two CASPR2-antibody patients’ IgGs (CASPR2-IgGs) were shown to bind strongly to the surface of live cells expressing EGFP-tagged human CASPR2, to live (unfixed) rodent hippocampal neurons, and to sections of fixed mouse brain from wild-type mice. There was no binding to live hippocampal neurons or to brain sections from *Cntnap2* knockout mice (B6.129(Cg)-Cntnap2^tm1Pele^/J; Jackson Laboratory), a homozygous knockout mouse for the gene encoding CASPR2, confirming that there was absent or limited reactivity with other neuronal targets in the CASPR2-IgG preparations (Fig. 1a, top panels). Healthy control-IgG 1-3 did not bind to human CASPR2-EGFP-transfected cells, mouse live hippocampal neurons, or brain tissue sections (Fig. 1a, bottom panels). Plasma CASPR2-antibodies from both patients bound to wild-type fetal brain tissue at E18.5 (Fig. 1b). In the isocortex, human IgG from CASPR2-antibody patients was detected around the neuronal cell body and dendritic processes, extending radially to the deeper layers, as already reported for CASPR2 expression [11]. This staining was absent in the *Cntnap2* knockout mice when CASPR2 or HC plasma was used.

### Animals

Crl: MF1 outbred female mice (20–25 g) and adult male breeders of the same strain were purchased from a licensed breeding establishment (Charles River, Kent, UK Ltd). The animals were housed and mated in the Biomedical Services Unit at the John Radcliffe Hospital under standard laboratory conditions (ad libitum access to food and water; 12:12 light:dark cycle with lights on at 7 am). Establishment of pregnancy relied on detection of vaginal coagulant plugs the morning after mating. E0.5 was defined as the day on which the plug was detected and P0 as day of delivery. All in vivo experiments, with IgG preparations coded and investigators blinded, were performed in accordance with the United Kingdom Home Office Animals in Scientific Procedures Act (1986) and in accordance with institutional guidelines under HO PPL 40/3581.

### Experimental design

**Fig. 2:**
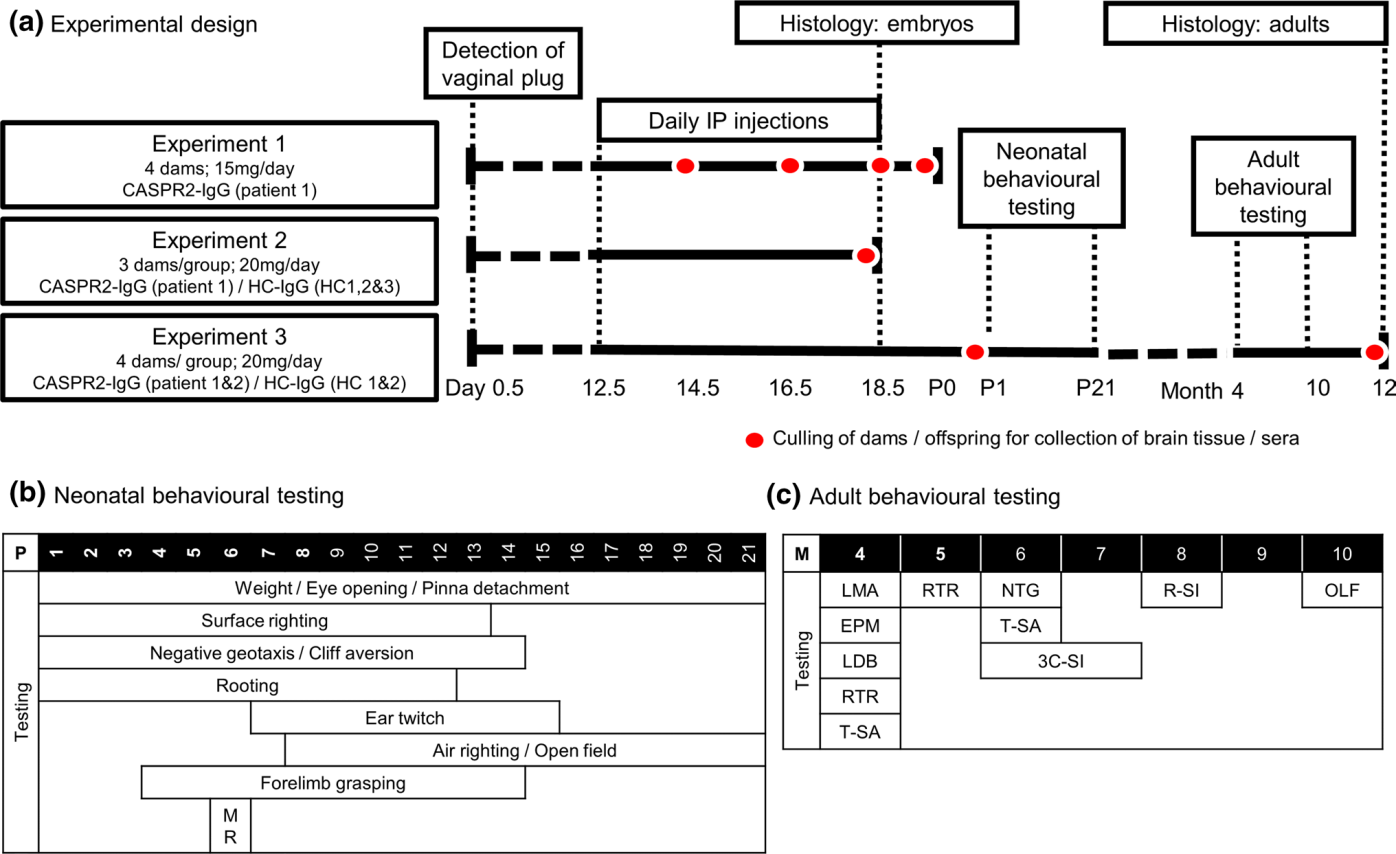
Experimental design and behavioral testing. **a** Experimental design. Experiment 1: Crl:MF1 dams (*n* = 4) were injected intraperitoneally (IP) with 15 mg of CASPR2-IgG daily from E12.5. One dam and offspring were sacrificed at each time point (E14.5, E16.5, E18.5, and P0) and sera collected for human IgG quantification and CASPR2-antibody testing. Experiment 2: Crl:MF1 dams (*n* = 3/group) were injected IP with 20 mg of the two CASPR2-IgGs and three HC-IgGs or equal volumes of saline from E12.5 until E17.5. All dams and offspring were sacrificed at E18.5 and embryonic tissue collected for analysis. Experiment 3: Crl:MF1 dams (*n* = 4/group) were injected IP with 20 mg of two CASPR2-IgGs and two HC-IGs from E12.5 until E18.5. At P1, litters were reduced to 6 pups. Neonatal assessment was conducted from P1 to P21 and adult assessment from 4 to 10 months of age. At 12 months, animals were sacrificed and brains dissected for histology. **b** Behavioral assessment. Neonatal testing performed as indicated in the table and continued daily until pup reached criterion on 2 consecutive days. The maternal retrieval (MR) test was performed at P6. Testing was done in all pups [*n* = 24 (4 litters)/group]. **c** Adult testing performed as indicated, and performed in half of the cohort (12 animals/treatment group), with the exception of the locomotor activity and the T-maze spontaneous alternation test done in all animals (1 HC-IgG mouse deceased for non-experimental reasons), and the reciprocal social interaction test done in all pairs meeting weight-matching criteria (*n* = 15 CASPR2-IgG pairs; *n* = 6 HC-IgG pairs). *LMA* locomotor activity, *EPM* elevated plus-maze, *LDB* light–dark box, *RTR* accelerating rotarod, *T-SA* T-maze spontaneous alternation, *NTG* nesting, *3C-SI* 3-chamber social interaction, *R–SI* reciprocal social interaction, *OLF* olfactory test

There were three main experiments (Experiment 1–3). In each, dams were tagged and randomized between cages, and injected intraperitoneally with independently coded purified human IgG preparations (15–20 mg/day; volume 1–2 ml) daily from E12.5 until E18.5 or until the time of culling [15], as indicated in Fig. 2a.

Experiment 1 assessed the maternal-to-fetal transfer of antibodies. Fifteen milligrams of one CASPR2-IgG was injected daily into four pregnant dams from E12.5 onwards. Dams were sacrificed at E14.5, E16.5, E18.5, and P0 (one dam per time point) by CO_2_ anaesthesia, followed by cardiac puncture for blood collection. The dams were then dissected and the uteri removed. Fetuses were dissected out of their membranes, separated from the placenta, and washed in saline. Fetuses/pups were sacrificed by decapitation, and fetal blood was collected by suspending the trunk. Blood was pooled from the fetuses/pups of each litter and the sera separated and stored at −20 °C. Total human IgG was determined by quantitative western blotting and CASPR2-antibodies detected by a live cell-based assay as above (details in Online Resource 3).

Experiment 2 examined the embryos at E18.5 after injection of three dams per group with 20 mg IgG per day. Dissected fetuses were decapitated and flash frozen in ice-cold isopentane. Twelve micrometer sagittal sections of the entire head were obtained from 3 fetuses per treatment group, from 3 different litters, and used for immunohistochemistry (IHC)/immunofluorescence. Details are provided in Online Resource 3.

Brain sections (1 in 10 series) from one embryo per litter were stained for total human IgG. In the same slide containing 4 consecutive sections, the two bottom sections were briefly washed 3 times in phosphate buffer saline (PBS), while the two upper sections were unwashed. All sections were then fixed with 4% paraformaldehyde (PFA) for 10 min. After 3 washes in PBS, sections were incubated with CF488A anti-human IgG (Biotium, 20022) at 1:500 overnight at 4 °C. The following day, sections were washed 3 times in PBS and incubated with rabbit anti-von Willebrand factor (VWF; Millipore, AB7356, 1:250) for 3 h at room temperature (RT), followed by incubation with goat anti-rabbit Alexa Fluor 568 secondary for 1 h at RT. After washing 3 times in PBS, coverslips were mounted using fluorescent mounting media containing DAPI (1:1000). Sections were visualized using a Leica DM 2500 immunofluorescence microscope. For quantitative analysis of the mean fluorescent intensity, 3 photomicrographs were taken from the isocortex in 3 different sections from 3 embryos/treatment group (27 photomicrographs/treatment group). Mean fluorescence intensity was then assessed for each photomicrograph using ImageJ. Results were plotted for both individual photomicrographs and for the mean results from each pup.

Human IgG was eluted from CASPR2-IgG and HC-IgG exposed embryos (3 embryos per group) following a previously described protocol [6] and as detailed in Online Resource 3.

Experiment 3 looked at the long-term effects of maternal–fetal transfer. Dams were injected daily from E12.5 to E18.5 with CASPR2-IgG from two patients and HC-IgG from two donors (4 dams per treatment group; IgG concentration 20 mg/day; volume 1–2 mL/day) and allowed to deliver. At P0 (usually the morning following delivery), the number of live and dead pups was counted and the litter left undisturbed with the dam for 24 h. At P1, to ensure equal maternal interactions, six pups were randomly selected from each litter and individually tattooed for coded behavioral testing. During these procedures, the dam was left in the home cage and was placed in the testing room for habituation. The remaining pups from that litter were culled by decapitation and blood was pooled for antibody testing.

### Behavior analysis of the offspring

Behavioral experiments were conducted at the times indicated in Fig. 2b and c. Behavioral testing of the neonatal pups was performed from postnatal days 1–21 and consisted of evaluation of developmental milestones using the modified Fox battery [12], which assesses physical maturation readouts, neurological reflexes, and motor coordination, and a maternal retrieval test, as described in Online Resource 3.

A battery of behavioral tests was performed in the mice when adults (>4 months old, mainly 4–8 months). Tests performed assessed locomotor activity, motor coordination, anxiety, short-term memory, social interaction/activities and non-social behaviors, and olfaction (see Online Resource 3). All behavioral tests and subsequent histological analyses were performed blinded to treatment group.

### Morphological and morphometric analyses

After behavioral testing, six animals per treatment group (3 males and 3 females) were randomly selected for histological analysis at 12 months. Mice were deeply anaesthetized with Isoflurane followed by pentobarbital injection (100 mg kg^−1^ ip) and transcardially perfused with 100 ml of PBS followed by 50 ml of ice-cold 4% PFA in 0.1 M phosphate buffer. Brains were removed and post-fixed for 24 h at RT before transferring to 30% sucrose in PBS. Using a freezing stage sledge microtome, free-floating coronal sections were obtained in 15 series at a thickness of 50 μm/section.

The detailed methods for cortical layer measurements and immunohistochemistry for neurons and microglia are given in Online Resource 3. CUX-1-positive cells in layers V and VI of the somatosensory cortex were counted in the coronal slices at Bregma—1.46 mm. 16 images (418.4 μm × 418.4 μm) were taken per mouse using a Zeiss confocal microscope in one plane (depth = 5 μm) on 50 μm coronal sections incubated in fluorescently tagged antibodies. At least 350 cells were counted per brain using Fiji cell counter and an average density (cells/mm^2^) per case was obtained.

Quantification of activated microglia (defined as CD68/Iba-1 positive cells) was performed in layers I, II–III, and V–VI in the infralimbic and prelimbic areas (Bregma +1.42 mm) and in layers I, II–IV, and V–VI in the somatosensory cortex (Bregma −1.46 mm). The z-stack area was 174.3 μm × 174.3 μm for the prefrontal areas and 111.15 μm × 111.15 μm for the somatosensory areas. The z-step/interval was 2 µm and microglial cells were counted within a 50 µm depth. Four z-stacks per layer were imaged in the infralimbic and prelimbic areas and six z-stacks per layer in the somatosensory area. An average density was obtained (cells/mm^3^) in both hemispheres. At least 100 cells per layer were counted.

Microglial morphology was assessed in 24 confocal z-stacks/case at 40× magnification (28 μm thickness with imaging every 2 μm) detecting fluorescence in IBA-1 expressing cells in layers of the somatosensory cortex. Minimum resolution of 512 × 512 was used and with a line averaging of 4 to allow for a detailed assessment of microglial cells in each group. A macro (details in Online Resource 3) written for use in Fiji (Image Analysis software) was used to obtain in each stack a clear representation of the cell body and processes of 101 microglial cells per group. Soma size (μm^2^) and number of ramifications per cell were recorded manually in Fiji.

Immunohistochemistry for synaptic proteins was performed using a guinea pig anti-synaptophysin antibody (Frontiers Institute, Syn-GP-Af300; 1:200) and a rabbit anti-PSD95 antibody (Frontiers Institute, PSD95-Rb-Af628; 1:200) to identify the presynaptic zone and the post-synaptic density of asymmetric glutamatergic synapses, respectively. For GluA1/PSD95 immunofluorescence, a rabbit anti-GluA1 antibody (Frontiers Institute, GluA1-Rb-Af692; 1:200) and a guinea pig anti-PSD95 antibody (Frontiers Institute PSD95-GP-Af660; 1:200) were used. 50 μm sections were air-dried for 48 h, rehydrated in PBS for 15 min, and then placed in a solution of 50% ethanol: PBS for 30 min at RT. Sections were subsequently dipped in double distilled H_2_0 to wash off the ethanol then in citrate-EDTA buffer (10 mM citric acid, 2 mM EDTA, 0.05% Tween-20 at pH 6.2) prior to a heat-mediated antigen retrieval step in citrate buffer for 10 min. Proteinase K treatment (4 μg/ml) for 10 min followed by a 10 min pepsin treatment (1 mg/ml) at 37 °C in 0.2 M HCl solution were required to unmask antigenic epitopes. Sections were then incubated with primary antibodies diluted in PBS-Azide-Triton-X-100 (0.3%) for 3 days at RT, and washed and incubated overnight with anti-guinea pig (1:500) and anti-rabbit (1:500) secondary antibodies from Life Technologies conjugated to Alexa Fluor 555 and 488 fluorophores, respectively, and concentrated DAPI at 1:50,000 in PBS. Sections were subsequently washed and mounting medium added. Two z-stacks measuring 66.11 × 66.19 × 6 μm with a voxel size of 0.04 × 0.04 × 0.1 μm (XYZ) were taken in each layer of the infralimbic, the prelimbic, and the somatosensory cortices in both hemispheres. This resulted in 60 optical sections available to quantify the number of PSD95-positive profiles and GluA1-positive profiles (synaptic contained in PSD95-positive profiles and extra-synaptic negative for PSD95) and at least 2000 puncta per layer were quantified. All image processing and analyses was performed in Fiji. To process images for quantification of synaptic profiles, first z-stacks were deconvolved using the WPL deconvolution algorithm in the Parallel Iterative Deconvolution ImageJ plugin [38] based on the Iterative Deconvolve 3D algorithm [27] utilizing a theoretical 3D point spread function generated in PSF Lab [19]. Image stacks were then filtered with a 3 × 3 median filter and thresholded using the OTSU method [20] to obtain accurate binary representations of positive profiles. To assess synapse profiles, individual z-slices separated by 0.4 μm were isolated and positive puncta profiles were analyzed using the Analyze Particles algorithm in Fiji. Puncta profile counts in each layer were averaged over two sections per animal, and plotted as profile counts per 100 μm^2^.

NeuN-positive cells were quantified by layer in the coronal slice at Bregma—1.46 mm. The z-stack area was 174.3 μm × 174.3 μm for the medial prefrontal areas and 111.15 μm × 111.15 μm for the somatosensory areas. The z-step/interval was 5 μm and NeuN-positive cells were counted within eight optical sections through the 50-μm stack. Four z-stacks per layer were imaged in the infralimbic, prelimbic, and somatosensory areas. An average density was obtained (cells/mm^3^) in both hemispheres. For layer I, at least 100 cells were counted; for the remaining layers, at least 400 cells/layer were counted.

### Statistics

Results were analyzed by independent samples *t* test or a Mann–Whitney *U* test, depending on data distribution, or by repeated-measures/two-way ANOVA. A Holm–Sidak correction was applied to correct for multiple comparisons when appropriate. A statistically significant result was considered if *p* < 0.05. Analysis was performed in IBM SPSS statistics version 21.0 (SPSS Inc., Chicago, USA). Graphs were plotted using Graph Pad prism version 6 (GraphPad software, San Diego, California, USA). To account for intra-litter effects during the neonatal period, the means for each litter were used in statistical comparisons, rather than the data from individual pups. In adult animals, unless mentioned otherwise, data represent the mean ± SEM of average values derived from individual animals.

## Results

### Human IgG and specific CASPR2-antibodies are transferred from maternal to fetal circulation and brain parenchyma (Experiment 1 and 2)

**Fig. 3:**
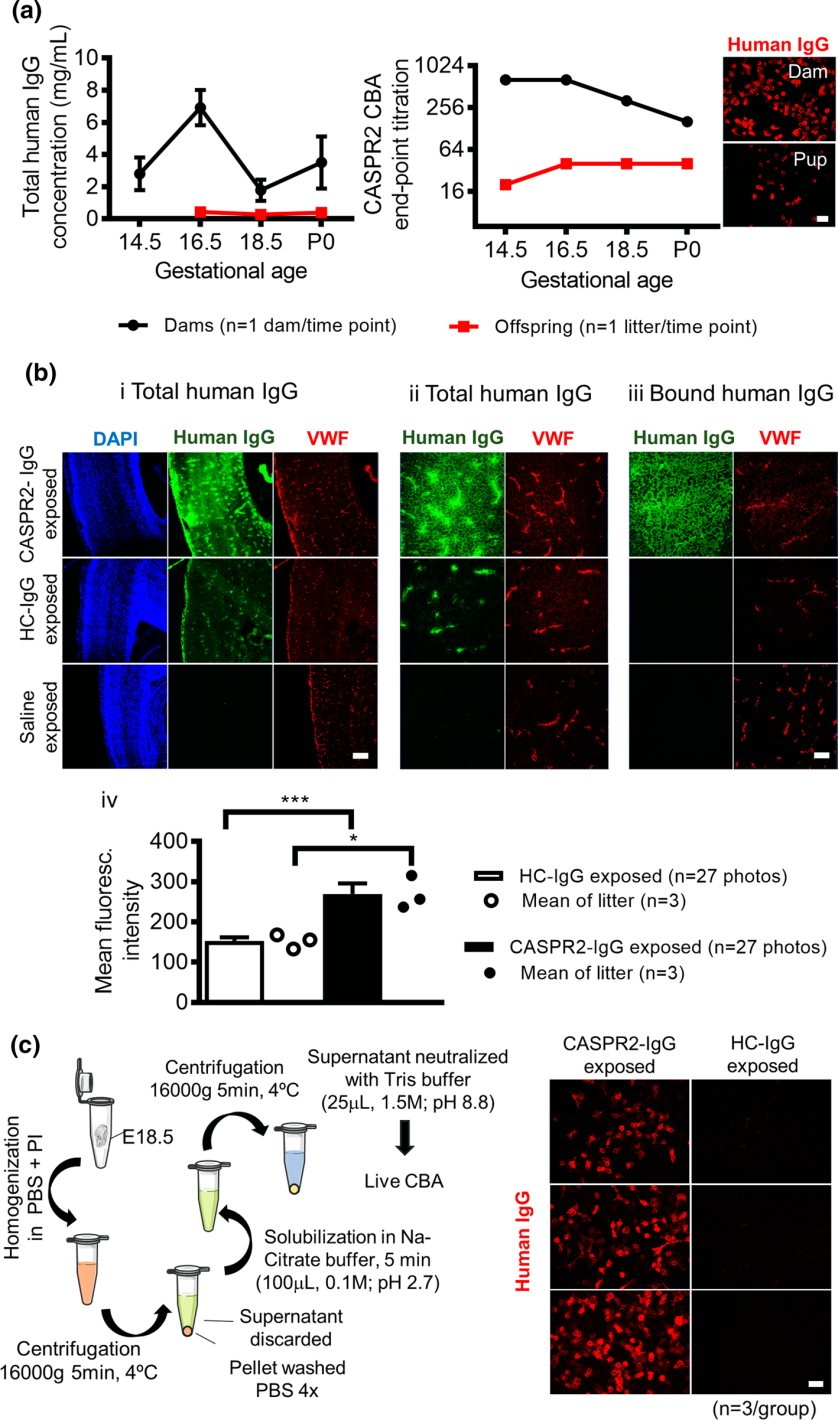
Transfer of human IgG/CASPR2-antibodies from maternal to fetal circulation and brain parenchyma. **a** Total human IgG was measured by western blotting in dams and fetus/pups sera at several gestational time points (1 dam per time point) after injection of human IgG daily (15 mg/day; titer 1:6400) from E12.5. Data presented as mean ± SEM of three western blot replicates (*left panel*). CASPR2-antibody levels in sera from dams and embryos/pups throughout gestation and representative images of the cell-based assay testing for CASPR2-antibodies in sera from dam and pups at P0 (human IgG, *red*) (*right panel*). Scale bar: 25 µm. **b** *i* Total human IgG (*green*), in fixed sections of embryonic brain (isocortex), co-localizes with a vessel marker, Von Willebrand factor (VWF, *red*), in the CASPR2-IgG and HC-IgG exposed but not in saline-exposed embryos. Scale bar: 200 µm. *ii* Higher magnification showing that human IgG is also visible beyond the vasculature in the parenchyma of CASPR2-IgG and, to a lesser extent, HC-IgG exposed embryos. *iii* Human IgG is still detected in the embryonic parenchyma after gentle wash of unbound IgG in the CASPR2-IgG but not in the HC-IgG exposed litter. *Scale bar*: 50 µm. *iv* Amount of bound human IgG (washed slides) in the isocortex, determined by the mean fluorescence intensity analysis of embryonic brain sections, showing significantly higher intensity in the CASPR2-IgG exposed litter. Data presented as mean ± SEM. **c** Elution of antibodies bound to the parenchyma of E18.5 CASPR2-IgG and HC-IgG (*n* = 3 animals/group) was performed as depicted in the figure (*left panel*) and the elution supernatant tested on a live human CASPR2 cell-based assay (*right panel*). CASPR2-antibodies were detected in the eluate from CASPR2-IgG but not from HC-IgG exposed embryos (anti-human IgG, red). *Scale bar*: 25 µm

The maternal human total IgG levels varied between 3 and 7 mg/ml over the 7 days of transfer (E12.5–E18.5), while human IgG in the fetal sera did not exceed 0.4 mg/ml at the end of gestation (i.e., about 10% of maternal levels at that time; Fig. 3a, left panel). Nevertheless, the CASPR2-antibodies were clearly present in the pool of fetal sera, with titers at the end of gestation of 1:40 in Experiment 1 (Fig. 3a, left panel) and 1:100–1:200 in the pups in Experiments 2 and 3 (data not shown), where higher titer IgG preparations were used.

To see whether human IgG could be detected in the brain tissue of the fetal brains shortly before delivery (Exp 2), three dams per treatment group were injected with HC-IgG or CASPR2-IgG (patient 1, 20 mgs/day) from E12.5 to E17.5, and the dams culled at E18.5. Visual inspection of all embryos prior to culling showed no gross malformations in the litters of either treatment group, and there were no gross brain morphological abnormalities on Nissl staining of the embryo heads after snap-freezing (Online Resource 4).

When the fetal brain sections were PFA-fixed on the slide, intense deposition of human IgG was observed in the fetal vessels [co-labeled with anti-von Willebrand factor (VWF) antibody], choroid plexus, and the periventricular zones in both experimental groups (Fig. 3b, panel i), as expected from circulation of the IgG. However, there was greater deposition of human IgG in the perivascular areas of the CASPR2-IgG than in the HC-IgG exposed fetuses suggesting that some might be bound in situ (Fig. 3b, panel ii). This was confirmed when adjacent sections were washed gently to remove unbound IgG before fixation; in CASPR2-IgG—but not HC-IgG exposed brains—human IgG was clearly bound in the parenchyma (Fig. 3b, panel iii). The lack of detectable bound IgG in three saline-exposed offspring, on either fixed or unfixed tissue, confirmed the specificity of the binding for human IgG (Fig. 3b, i–iii).

These observations were quantified by measuring the mean fluorescence intensity in the isocortex. Despite some variability between sections of the same embryo, the fluorescence intensity in the CASPR2-IgG exposed embryos was higher than in the HC-IgG exposed embryos, considering either the mean fluorescence intensity of all photomicrographs [*t*(33.2) = 4.2, *P* < 0.0001] or the mean for each embryo [*t*(4) = 4.54, *P* = 0.01; Fig. 3b, panel iv]. Thus, IgG remained bound to the brain tissue in the CASPR2-IgG exposed. To establish whether this bound IgG was directed against CASPR2, acid elution of the bound human IgG was performed (*n* = 3 animals/group), following a previously described method [6] (Fig. 3c). CASPR2-antibodies were detected in the eluate from CASPR2-IgG but not from HC-IgG exposed embryos. However, at this gestational age, a reduction in CASPR2 expression was not detected in the CASPR2-IgG exposed embryos in comparison with HC-IgG exposed embryos by western blotting (Online Resource 5).

### Neurodevelopmental milestones after intrauterine exposure to CASPR2-IgG (Experiment 3)

**Fig. 4:**
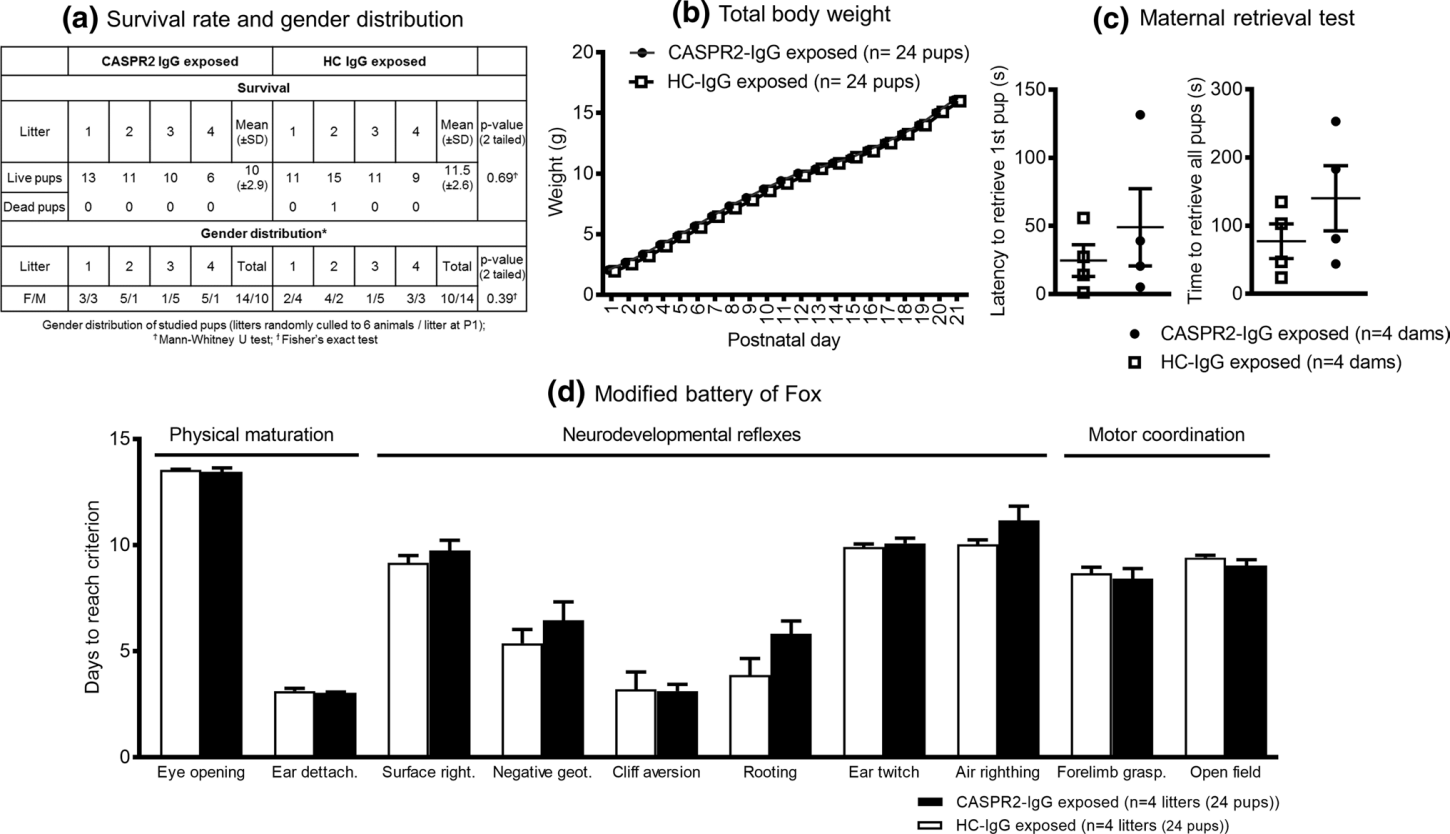
Neonatal period assessment of mice exposed to CASPR2-IgG and HC-IgG in utero. **a** No significant differences in the survival/gender distribution of CASPR2-IgG versus HC-IgG exposed pups. Gender distribution determined after random culling of litters to 6 pups/litter. **b** Total body weight during neonatal development of CASPR2-IgG and HC-IgG exposed offspring showed no differences (*n* = 24 pups/group; group *P* = 0.6; group × time *P* = 0.51, repeated-measures ANOVA). **c** No effect of treatment on the time taken by the mother to return all pups to the nest (*P* = 0.46, independent samples *t* test) or latency to retrieve first pup (*P* = 0.29, independent samples *t* test; *n* = 4 dams/treatment group). **d** Neonatal development assessed by the modified Fox battery in the readouts of physical maturation, neurodevelopmental reflexes, and motor coordination tests showed no differences between groups, [4 litters (24 pups)/treatment group]; (*P* > 0.05, independent samples *t* test). Data presented as mean ± SEM

For behavioral testing, four dams per treatment group were injected daily (two with patient 1 and two with patient 2) from E12.5 to E18.5 and allowed to deliver. HC-IgG and CASPR2-IgG exposed litters showed no differences in the number of surviving pups (U = 6; Z = −0.59; *P* = 0.69) and no obvious gross abnormalities. To ensure that the behavioral results were not influenced by different litter sizes, which could affect maternal–offspring interactions, each litter was randomly culled to 6 mice at P1. Gender of the randomly selected pups for study did not differ between groups (Fisher’s exact test *P* = 0.39; Fig. 4a).

There was no difference in weight increase over the postnatal period (group × time *F*(2.8, 127.2) = 0.27, *P* = 0.51, Greenhouse–Geisser correction; group *F*(1,46) = 0.28, *P* = 0.6; Fig. 4b). The maternal retrieval test, which assesses maternal-infant interactions, showed variation but no differences between groups in the time taken by the dam to return all pups to the nest [*t*(6) = 1.2, *P* = 0.46], or in the latency to retrieve the first pup [*t*(6) = 0.8, *P* = 0.29; Fig. 4c]. There were no differences in any of the parameters analyzed by the modified Fox battery, including physical maturation readouts, neurodevelopmental reflexes, or the motor coordination performance (Fig. 4d); a modest increase in days to reach rooting behavior was not significant.

### Adult behavior after intrauterine exposure to CASPR2-IgG (Experiment 3)

**Fig. 5:**
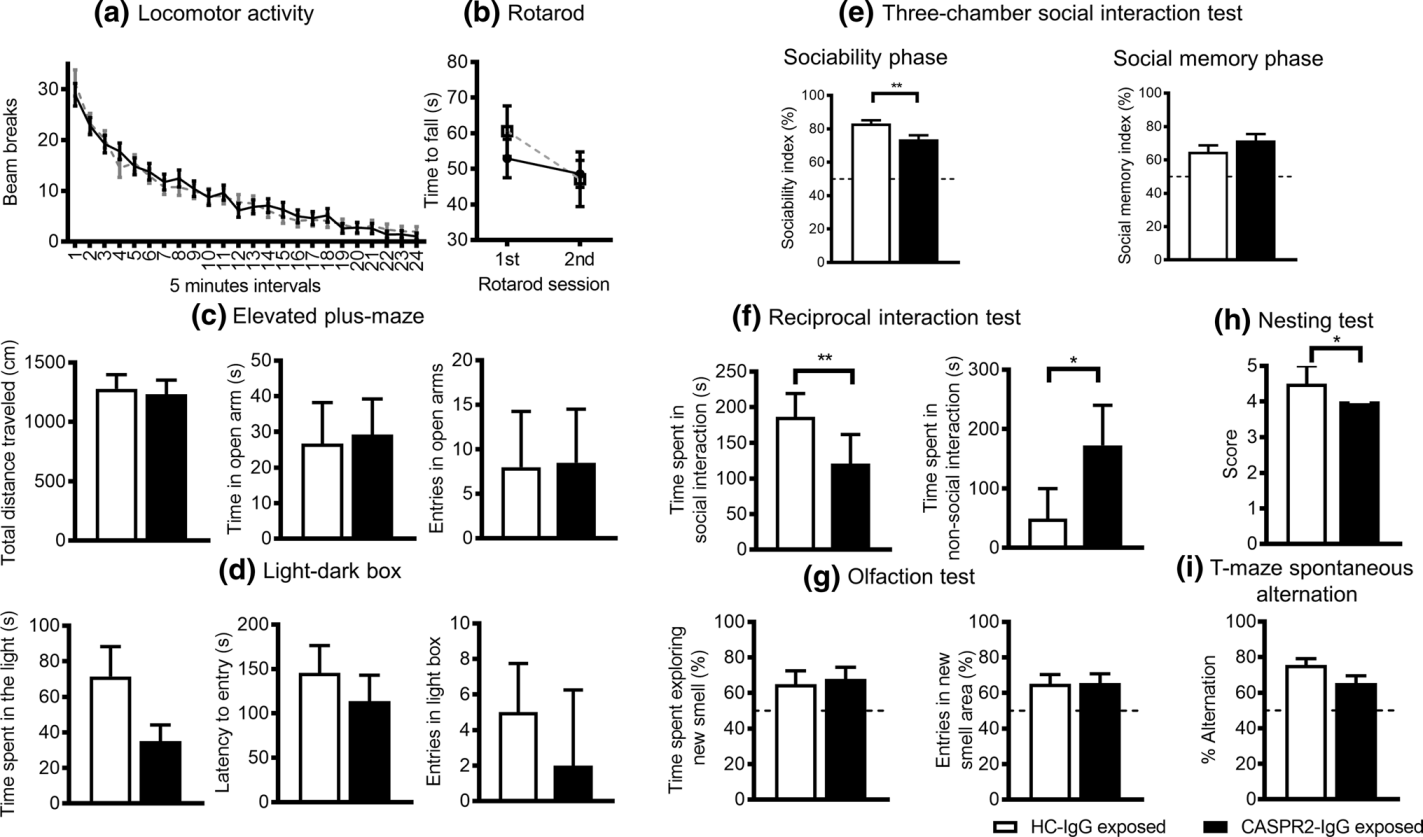
Behavioral testing of adult mice exposed to CASPR2-IgG and HC-IgG in utero. **a** Locomotor activity: No differences between groups (*n* = 24 CASPR2-IgG/23 HC-IgG; group *P* = 0.23; time × group *P* = 0.91, RMANOVA). **b** Accelerating rotarod: no differences between groups in 2 rotarod sessions (*n* = 12/group; group *P* = 0.7; session × group *P* = 0.27, RMANOVA). **c** Elevated plus-maze: no differences between groups in total distance travelled (*P* = 0.79, independent samples *t* test), time in open arms (*P* = 0.87, independent samples *t* test) or entries in open arms (*P* = 0.89, Mann–Whitney test; *n* = 12/group), and test duration: 300 s. **d** Light–dark box: no differences between groups for time in light box (*P* = 0.07, independent samples *t* test), latency to enter light box (*P* = 0.46, independent samples *t* test) and number of entries in light box (*P* = 0.51, Mann–Whitney test; *n* = 12/group), test duration: 300 s. **e** 3-chamber social interaction test: CASPR2-IgG exposed mice spent significantly less time in social interaction during the sociability phase (*n* = 12/group; *P* = 0.006, independent samples *t* test), but no differences were found during the social memory phase (*P* = 0.22, independent samples *t* test). Sociability index was calculated as (the time spent in the interaction zone around the stimulus animal) divided by (the time spent in the interaction zone around the stimulus animal plus the time spent in the analogous zone of the empty chamber), and values presented as a percentage. Social memory index was calculated as (the time spent in the interaction zone around the novel animal) divided by (the time spent in the interaction zone around the novel animal plus the time spent in the analogous zone of the known animal), and values presented as a percentage. **f** Reciprocal social interaction test: CASPR2-IgG exposed mice engaged in less social interaction (*P* = 0.008; Mann–Whitney test) and spent more time in non-social activities (grooming/digging) than HC-IgG exposed mice (*n* = 15 pairs CASPR2-IgG/6 pairs HC-IgG; *P* = 0.04; Mann–Whitney test). **g** Olfaction test: no differences between groups in the preference for a novel odor, determined by percentage of time spent with the novel/familiar odor (*P* = 0.78, independent samples t test) or percentage of entries in the novel/familiar odor interaction zone (*P* = 0.95, Mann–Whitney test). **h** Nesting test: CASPR2-IgG exposed mice show decreased nesting behavior (12/group, *P* = 0.03; Mann–Whitney test). **i** T-maze spontaneous alternation: marginal effect with reduced alternation in the CASPR2-IgG exposed group (*n* = 24 CASPR2-IgG/23 HC-IgG, *P* = 0.067, independent samples *t* test). Data presented as mean ± SEM, except number of entries in *open arms*, number of entries in *light box*, and reciprocal interaction test presented as median with interquartile range; * *P* < 0.05; ** *P* < 0.01

The mice from Experiment 3 were maintained in their cages and studied between ages 4–10 months. There were no significant differences in tests of locomotor activity (Fig. 5a), motor coordination (accelerating rotarod; Fig. 5b) or in anxiety tests (elevated plus-maze and light–dark box; Fig. 5c/d), although a trend towards less time spent in the light was noted in the CASPR2- IgG exposed mice (*P* = 0.07; Fig. 5c/d).

Social behavior was assessed using two methods at 6–8 months. In the three-chamber social interaction test, both CASPR2-IgG and HC-IgG exposed mice showed a preference to spend time exploring the wire cage containing a novel, unfamiliar mouse compared with an empty wire cage, but CASPR2-IgG exposed mice spent significantly less time interacting with the mouse than HC-IgG exposed mice [*t*(22) = 3.086, *P* = 0.006; raw data available in Online Resource 6]. When subsequently given a choice between the same mouse and a new mouse, both CASPR2-IgG and HC-IgG exposed mice preferentially explored the novel animal suggesting no social memory deficits [*t*(22) = 1.253, *P* = 0.223; Fig. 5e]. Using the reciprocal social interaction test, CASPR2-IgG exposed mice were again found to spend less time engaged in active social interaction (sniffing, licking, mounting, or crawling under or over the other mouse; Fig. 5f) than the HC-IgG exposed mice (*U* = 11, *Z* = −2.647, *P* = 0.008), confirming a significant deficit in social interaction in those mice exposed to CASPR2-IgG in utero.

Importantly, there were no differences between groups in measures of locomotor activity during the period of habituation to the testing arena, ensuring that the decrease in social interaction was not due to differences in exploratory behavior or habituation to the testing environment (Online Resource 7). Furthermore, there were no differences in an olfactory test, with both groups showing an equivalent preference for a novel odor compared to a recently experienced, and thus familiar, odor (Fig. 5g). In addition, there were no differences between groups in the time spent exploring the odor area during the sample phase of this test [*t*(14) = 0.55, *P* = 0.6; data not shown]; these results confirmed that the social deficits observed were not due to reduced olfaction or response to novelty.

Non-social behaviors (grooming, digging) were also assessed during the reciprocal interaction test. CASPR2-IgG exposed mice spent more time engaged in those activities than HC-IgG exposed mice [*U* = 19, *Z* = −2.024, *P* = 0.04; Fig. 5f, right panel]. In a nest-building test, a species-typical behavior claimed to be potentially relevant to home–cage social interaction [23], CASPR2-IgG exposed mice scored less than the HC-IgG exposed animals [*U* = 39, *Z* = −2.126, *P* = 0.03; Fig. 5h]. All mice were also tested for spontaneous alternation in the T-maze, a test of short-term memory, and a trend towards a decrease in alternation was noted in the CASPR2-IgG exposed mice [*t*(45) = −1.874, *P* = 0.067; Fig. 5i]; this is of potential relevance considering that *Cntnap2* knockout mice were found to alternate less in the same test [23].

The results from all behavior tests were first analyzed without separating the genders. On further analysis (Online Resource 8), we found no gender effect in any of the behavioral parameters analyzed, with the exception of the nesting test [H(3) = 9.6, *P* = 0.02; Kruskal–Wallis test], which showed lower scores in CASPR2-IgG exposed males (*U* = 33, *P* = 0.01; Mann–Whitney test) but not in females (*U* = 18, *P* = 1.0; Mann–Whitney test). A trend towards a gender effect was also noted for the light–dark test and the time spent in non-social activities during the reciprocal social interaction test, with male CASPR2-IgG mice being more affected.

### Adult mice after intrauterine exposure to CASPR2-IgG show abnormal lamination of the somatosensory cortex (Experiment 3)

**Fig. 6:**
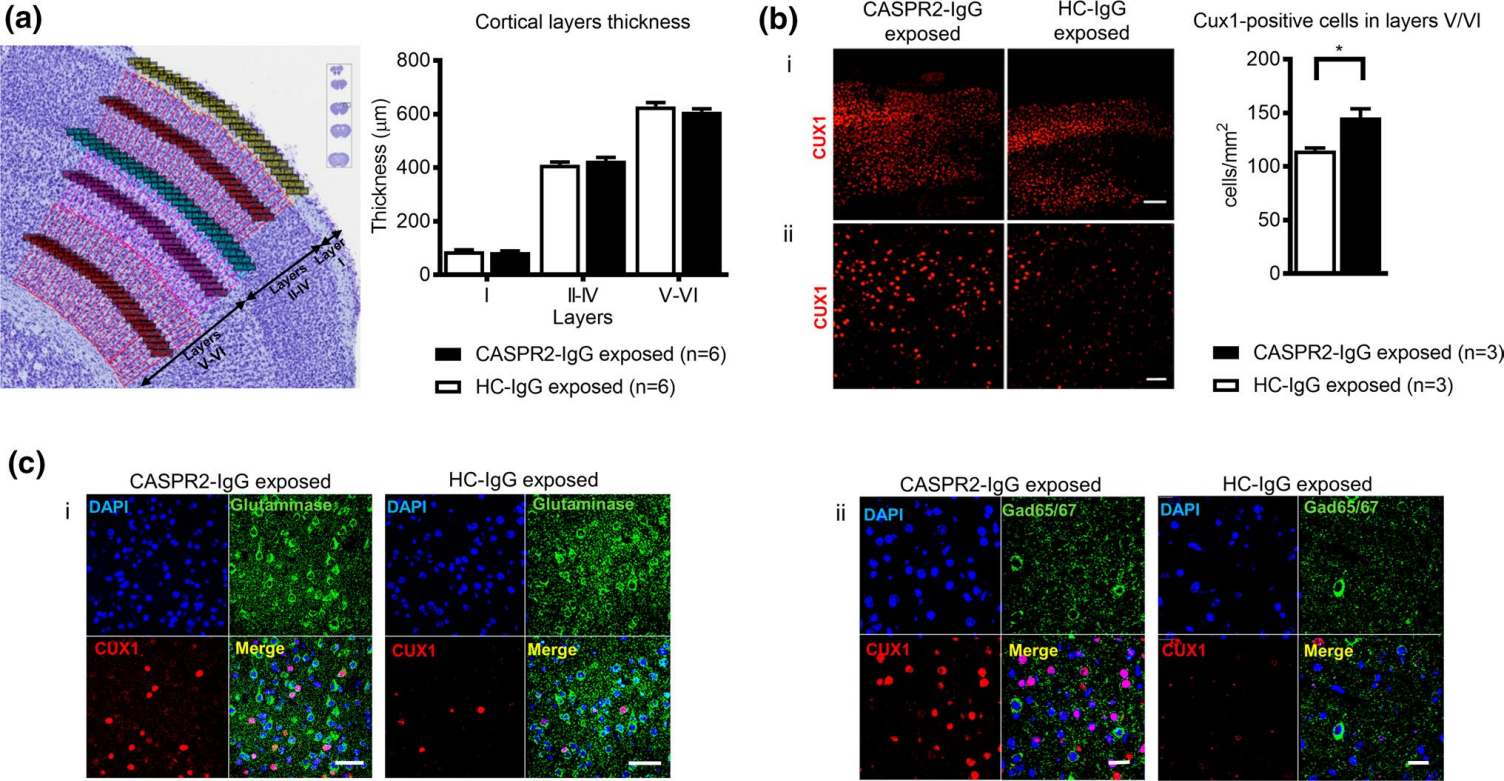
Lamination patterns of the somatosensory cortex. **a** Representative image of the histological boundaries used to define the different somatosensory cortical layers (*left*) and cortical thickness measurements (*right*), showing no differences found between CASPR2-IgG and HC-IgG exposed animals (*n* = 6 brains/group). **b** Representative images of the CUX1 (*red*) staining across treatment groups at low (*i*; Scale bar: 200 µm) and high magnification (*ii*; *scale bar*: 50 µm), focusing on layers V/VI (*left*). Quantification of CUX1-positive cells in layers V/VI of the somatosensory cortex (*right*) showing statistically significant increase in CUX1-positive cells in layers V–VI in CASPR2-IgG versus HC-IgG exposed animals (*n* = 3 brains/group). **c** Representative images of the *i* CUX1 (*red*)/glutaminase (*green*) and *ii* CUX1 (*red*)/GAD 65/67 (*green*) staining of neurons in layers V–VI in the somatosensory cortex. *Scale bars*: 50 µm. All data presented as mean ± SEM; *P < 0.05

At 12 months, coded brains from 3 to 6 animals in each group were randomly selected for histological analysis. In particular, since abnormalities of neuronal migration were reported in this brain region in the *Cntnap2* knockout model [23], the somatosensory cortex was evaluated for the presence of alterations in normal cortical structure. No gross morphological abnormalities and no morphometric differences were observed in the overall thickness of the cortex or in the thickness of the upper, middle, and deep layers, as assessed by Nissl staining (Fig. 6a).

Laminar position patterns, analyzed by assessing FOXP2-positive (marker of layer VI) neurons, did not reveal any differences between CASPR2-IgG and HC-IgG exposed groups (data not shown). However, there was an increase in the density of CUX1-positive cells (marker of layers II–IV) in layers V/VI in CASPR2-IgG exposed animals [*t*(4) = 3.63, *P* = 0.02], suggesting a neuronal migration defect (Fig. 6b).

To determine which neurons were expressing CUX1 in layers V/VI, the GABAergic and glutamatergic markers GAD65/67 and glutaminase, respectively, were used. In both CASPR2-IgG and HC-IgG exposed animals, most CUX1-positive neurons in layers V/VI were glutamatergic and none were identified as GABAergic (Fig. 6c).

There were no morphometric differences between groups with respect to the prefrontal cortices (anterior cingulate, prelimbic, and infralimbic cortices), corpus callosum, hippocampus or cerebellum, and no gross morphological abnormalities or neuronal ectopias/misalignments were detected in any of these brain areas (Online Resource 9 to 13).

### Adult mice after intrauterine exposure to CASPR2-IgG display increased densities of microglia and microglia with an activated morphological phenotype (Experiment 3)

**Fig. 7:**
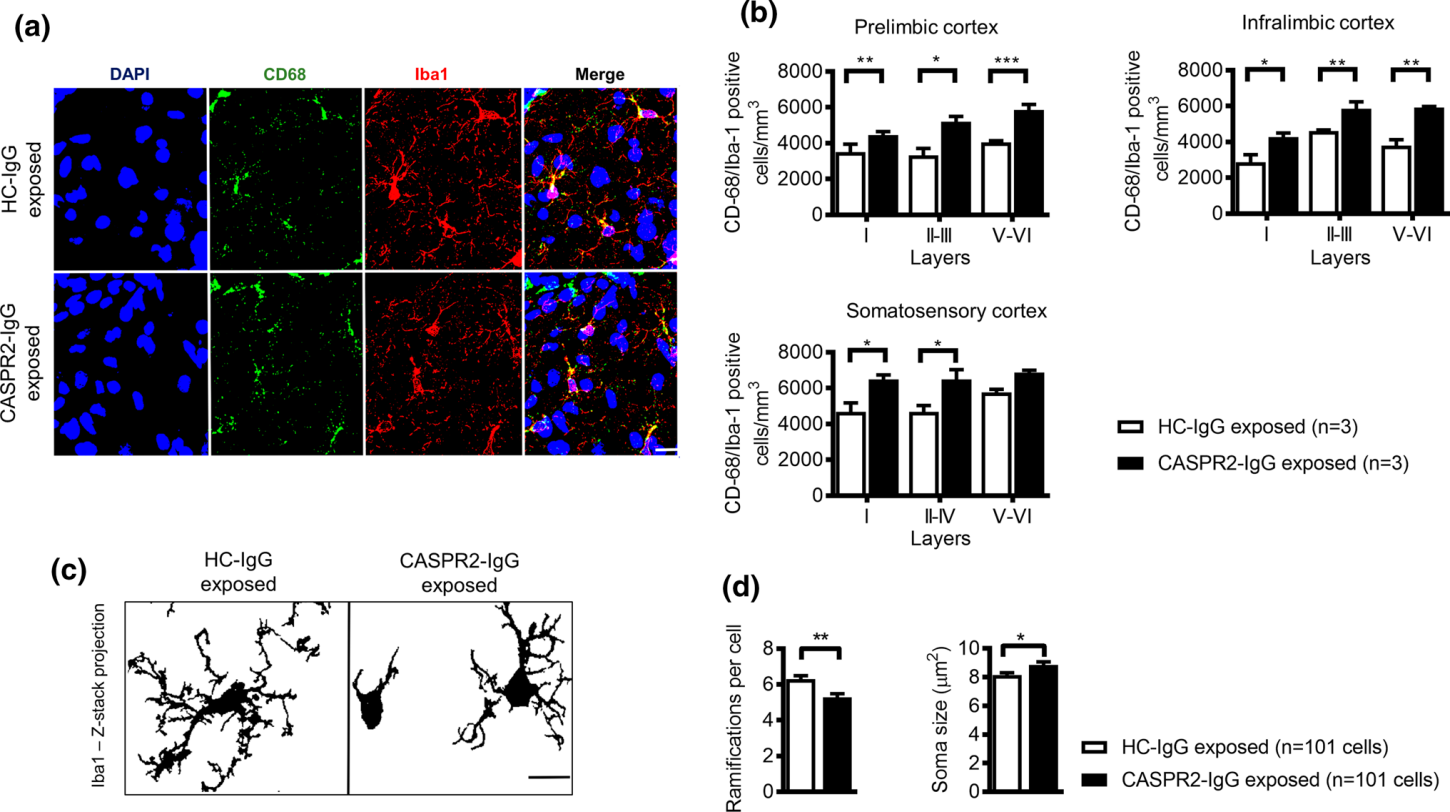
Activated microglia densities and morphological analysis. **a** Representative images of the somatosensory cortex showing microglial staining (DAPI, *blue*; CD68, *green*; Iba1, *red*). i: 25 µm. **b** Quantification of CD68/Iba1-positive microglial densities in the prelimbic, infralimbic, and somatosensory cortices (*n* = 3 brains/group). **c** Representative images of z-stack projections of Iba1-positive microglia used for morphological analysis. **d** Microglia morphological characterization: number of ramifications and soma size (*n* = 101 cells/group; 30–40 cells/animal; 3 animals/group). *P < 0.05; **P < 0.01; ***P < 0.001. All data presented as mean ± SEM

CD68/Iba1-positive cells (defining activated microglia) were quantified in the prelimbic, infralimbic, and somatosensory cortices. All *P* values below are adjusted according to the Holm–Sidak correction for multiple comparisons. There was an average 16% increase in mean Iba1/CD68-positive microglial densities in the CASPR2-IgG exposed animals, across all layers of the studied areas (Fig. 7a, b). In the prelimbic cortex, there was an increase in CASPR2-IgG exposed mice in all layers [layer I: *t*(12) = 2.2, *P* = 0.049; layers II–IV: *t*(12) = 4.3; *P* = 0.0032, layer V–VI: *t*(12) = 4.1; *P* = 0.0032), as also in the infralimbic cortex [layer I: *t*(12) = 3.5, *P* = 0.0095; layers II–III: *t*(12) = 3.0, *P* = 0.01; layers V–VI: *t*(12) = 5.2,* P* = 0.0007]. In the somatosensory cortex, the same trend was observed in all layers, statistically significant in layers I and II–IV [layer I: *t*(12) = 3.4, *P* = 0.014; layers II–IV: *t*(12) = 3.4, *P* = 0.014; layer V–VI: *t*(12) = 2.1, *P* = 0.054]. The results in embryos at E18.5 (Exp 2) were variable but showed a similar trend towards increased CD68-positive microglia (*P* = 0.01 uncorrected; Online Resource 14).

A detailed morphological analysis of parameters of microglial activation (number of ramifications and soma size) was performed in IBA-1-positive cells from the somatosensory cortex in both groups (*n* = 101 cells/group; 30–40 cells/animal; 3 animals/group) (Fig. 7c). Microglia in CASPR2-IgG exposed animals were significantly less ramified (*U* = 3.8; *P* = 0.001) and had increased soma size (*U* = 6.2; *P* = 0.014), consistent with an activated phenotype (Fig. 7d).

### Adult mice after intrauterine exposure to CASPR2-IgG display decreased densities of glutamatergic synaptic profiles in the prefrontal and somatosensory cortices (Experiment 3)

**Fig. 8:**
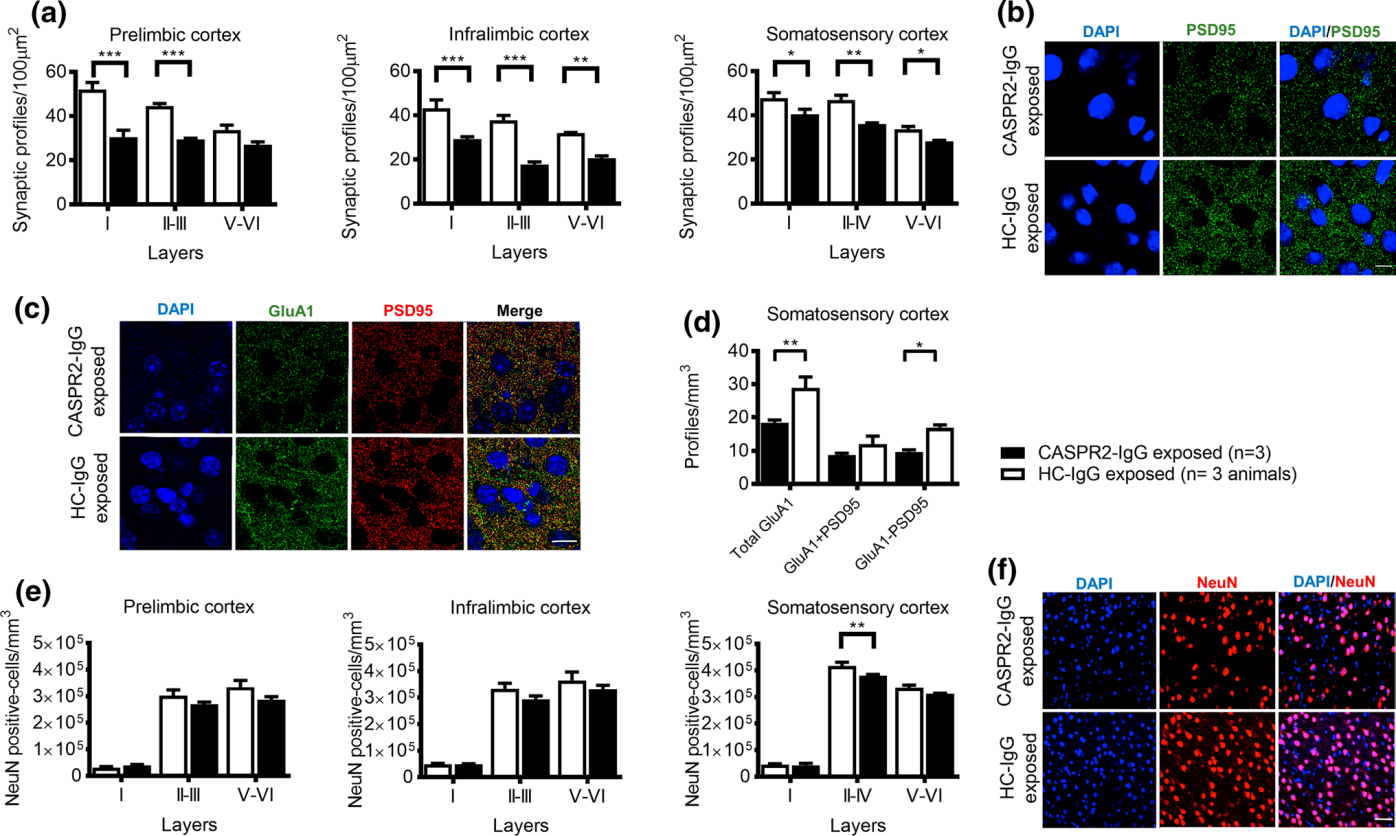
PSD95 and GluA1 profiles, and neuronal densities. **a** Quantification of PSD95-positive puncta in the prelimbic, infralimbic, and somatosensory cortices (*n* = 3 brains/group). **b** Representative images of the somatosensory cortex showing synaptic profile staining (PSD95, *green*; DAPI, *blue*). *Scale bar* 10 µm. **c** Representative images of the somatosensory cortex showing GluA1 and PSD95 staining (GluA1, *green*; PSD95, *red*; DAPI, *blue*). *Scale bar* 15 µm. **d** Quantification of Total GluA1 profiles, and synaptic (GluA1/PSD95-positive) and extra-synaptic (GluA1-positive/PSD95-negative) profiles in the somatosensory cortex. **e** Quantification of neuronal densities in the prelimbic, infralimbic, and somatosensory cortices (*n* = 3 brains/group). **f** Representative images of the somatosensory cortex showing neuronal staining (NeuN, *red*; DAPI, *blue*). Scale bar = 50 µm. *P < 0.05; **P < 0.01; ***P < 0.001. All data presented as mean ± SEM

It is well established that microglia play a role in synaptic pruning [22]. We identified the post-synaptic density of glutamatergic synapses using PSD95, confirmed by its association with the presynaptic terminal marker synaptophysin (Online Resource 15), and used a robust stereological sampling method to provide a large number of PSD95-positive profiles per layer (>2000 profiles). Our method covered a sufficient volume of each cortical layer to ensure sufficient sampling of the irregularly spaced PSD95-positive puncta in the neuropil and thus provided an accurate assessment of mean synaptic puncta density. We found a 15–52% reduction in the number of PSD95-positive synaptic profiles across the prelimbic, infralimbic, and somatosensory cortices in the CASPR2-IgG exposed animals (Fig. 8a, b). In the prelimbic cortex, the PSD95-positive synaptic profiles were reduced in layers I and II–III [layer I: *t*(12) = 7.0, *P* = 0.00004; layers II–III: *t*(12) = 4.9, *P* = 0.0007], with a strong trend in the deep layers [layer V–VI: *t*(12) = 2.1, *P* = 0.055]. In the infralimbic cortex, a significant decrease was seen in all layers [layer I: *t*(12) = 5.1, *P* = 0.0005; layers II–III *t*(12) = 7.3, *P* = 0.00003; layer V–VI *t*(12) = 4.1, *P* = 0.001], and also in the somatosensory cortex [layer I: *t*(12) = 3, *P* = 0.02; layers II–IV: *t*(12) = 4.5, *P* = 0.002; layer V–VI: *t*(12) = 2.2, *P* = 0.049]. These results demonstrate increased microglia and reduced glutamatergic synaptic profiles in the CASPR2-IgG exposed mice that were apparent as late as 12 months after birth.

Consistent with a decrease in glutamatergic synaptic profiles, CASPR2-IgG exposed mice had a significant reduction in the density of GluA1 profiles in the somatosensory cortex [*t*(12) = 4.2; *P* = 0.0036]. Both synaptic (GluA1/PSD95-positive puncta) and extra-synaptic (GluA1-positive/PSD95-negative puncta) GluA1 were numerically reduced in the CASPR2-IgG exposed mice, but this difference was significant only for extra-synaptic GluA1 profiles [*t*(12) = 2.9; *P* = 0.028] (Fig. 8 c, d). GluA1 expression was also examined in the embryos at E18.5, but, at this stage, no differences in total GluA1 expression were found between groups by western blotting (Online Resource 16).

Despite the reduction in synaptic profiles, the numbers of NeuN-positive neurons in the medial prefrontal cortex (prelimbic and infralimbic cortices) were not different between CASPR2-IgG and HC-IgG exposed animals (Fig. 8e, f). In the somatosensory cortex, there was a modest decrease in the neuronal densities in layer II–IV (*P* = 0.02), but not in layer I (*P* = 0.8) or layer V–VI (*P* = 0.1).

## Discussion

CASPR2-antibodies are now well recognized in patients with autoimmune forms of neurological disease, but have also been reported in a small subgroup of mothers (without any signs of overt disease) whose progeny were diagnosed with neurodevelopmental disorders [5, 8]. To clarify whether this association was related to CASPR2-antibodies or to other inflammatory/immune factors, we investigated how CASPR2-antibodies might mediate effects on the fetal brain using a previously established protocol for maternal-to-fetal transfer [15]. We injected patient IgG containing CASPR2-antibodies into pregnant mice and examined the offspring throughout the first year of life and post-mortem, comparing with IgG from healthy individuals. The mice exposed in utero to CASPR2-antibodies had positive serum levels and CASPR2-antibodies were bound to brain tissue, but neonatal behaviors were not affected. However, when examined as adults, the mice demonstrated altered social behavior accompanied by neuronal ectopias, reduced numbers of glutamatergic synaptic profiles, and increased densities of microglia with an activated morphology. Thus, this mouse study establishes that maternal autoantibodies to a CNS protein can cause long-term brain pathology and behavioral deficits. CASPR2-antibodies that we found raised in mothers of affected children [8] could contribute to neurodevelopmental deficits.

CASPR2 plays an important role during neurodevelopment [33], as demonstrated in vitro [1, 34] and in vivo [23]. A homozygous deletion of *CNTNAP2* in the mouse resulted in social deficits associated with disorganization of the lamination of the somatosensory cortex, aberrant arborization of dendrites, and maturation of dendritic spines with consequences for neuronal network activity. Other features of the *Cntnap2* knockout mouse, such as motor hyperactivity and hyper-reactivity to peripheral sensory stimuli, share some features with the immunotherapy-responsive peripheral nerve hyperexcitability and pain in adult patients with acquired CASPR2-antibodies [14]. Thus, in utero exposure to CASPR2-antibodies can lead to permanent changes in brain histology and behavior similar to those in the *CNTNAP2* disrupted mice, but do not reproduce the reversible, partly peripheral phenotype seen when adults are exposed to the same antibodies.

During the final stages of this study, others [5] also described CASPR2-antibodies in mothers of children with neurodevelopmental disorders, and studied a similar maternal-to-fetal transfer using a monoclonal CASPR2-antibody derived from one mother of a child with autism. They identified an ASD-phenotype, similar to that we observed here, but only in the male offspring of dams. We did not find a strong gender effect in our mice, although a trend to more affected males was observed in a few tests. It is possible that the differences between the gender results relate to the injected CASPR2-antibodies (a single injection of monoclonal antibody at E13.5 compared with daily injections of polyclonal patients’ IgG), timing of behavioral and histological assessments, or the choice of mouse strains; we deliberately used an outbred strain, Crl:MF1, because of their excellent maternal behavior (given the risk of maternal rejection due to early experimental manipulation of the pups) and large litters. Overall, our histological findings complement, rather than differ from those of Brimberg et al. who detected abnormalities in cortical development such as thinned cortical plate and decreased number of neural progenitors at E15.5, which might precede the abnormalities of cortical lamination we found in our animal model. Likewise, they reported a decrease in dendritic tree complexity and dendritic spine densities in the hippocampus, detected by Golgi stain, which is compatible with our findings of decreased density of glutamatergic synapses in the cortex in the adult offspring; in our mice, the hippocampal changes did not reach significance.

Aberrant glutamatergic synapse structure and function has been implicated in neurodevelopmental disorders such as autism, intellectual disability, and schizophrenia [37]. This theory is often based on the study of dendritic spine numbers and morphology in human post-mortem studies or in animal models of neurodevelopmental disorders [18]. Given that 90% of all excitatory synapses occur in dendritic spines, this is usually accepted as a useful proxy of synapse pathology. However, the results vary from increased spine densities observed in ASD and Fragile X syndrome to significantly decreased numbers of spines in Rett’s Syndrome, Down’s syndrome, or other forms of intellectual disability [24]. Moreover, the numbers and morphology of dendritic spines may not fully reflect the maturity of the glutamatergic synapse. Discrepancies in results could also arise from use of the Golgi method, which stains a very restricted population of neurons, thus producing a sampling bias. By contrast, here using a specific method that allows the quantification of synaptic profiles in a large volume of each cortical layer and a marker of mature glutamatergic synapses, PSD95 [25], the results pointed clearly towards a loss of glutamatergic synapses, with minimal neuronal loss, in layers of the medial prefrontal and somatosensory cortices in keeping with the role of CASPR2 in the establishment of stable excitatory synapses [1, 34]. In line with the glutamatergic synapse loss, we found a reduction in the AMPA receptor subunit GluA1, both total and extra-synaptic GluA1, consistent with the role of CASPR2 in GluA1 trafficking [34].

The inverse association between increased microglial densities per layer in the cortical areas examined and the associated decrease in synaptic profiles makes a compelling case for a role for microglia in pruning excitatory synapses in the CASPR2-antibody exposed mice. Synapse elimination occurs in the healthy developing brain and microglia are implicated as the key cellular players of this complement-mediated synaptic pruning. The microglia engulf synaptic material, a process that is partly mediated by the microglial C3 receptor and its ligand, the complement component C3 [22, 30] [32]. Supporting a role for microglia-dependent pruning in neurodevelopmental disorders, mice with decreased synaptic pruning due to genetic knockout of the chemokine receptor CX3CR1 (a crucial receptor for neuron-microglia signaling) displayed deficits in social interaction and increased repetitive behaviors [41]. However, this is, to our knowledge, the first link between activated microglia, synaptic defects, and any maternal autoantibody.

The most striking behavioral abnormalities identified were deficits in social interaction. These are usually interpreted in the literature as the hallmark of an autism-like phenotype, but in our study of gestational human cohorts [8], maternal CASPR2-antibodies were associated with mental retardation, speech and language impairments, and other undefined neurodevelopmental disorders, but not with autism *per se*. One must note, nevertheless, that impairment of social skills and stereotypies are symptomatic features common to patients with a range of neurodevelopmental disorders [7].

One can only speculate about the mechanisms by which CASPR2-antibodies cause changes in neurodevelopment. As discussed above, it is possible that mice exposed to these antibodies have an increased number of aberrant or weaker synapses that are then targeted for elimination. Alternatively, the antibodies may have bound directly to synapses to tag them for elimination by complement during development. Another major question relates to the persistence of microglia activation after an initial insult that occurs during development. It is now well accepted that an excess of reactive microglia is seen in the neuropathology of patients with different neurodevelopmental and neuropsychiatric disorders [35, 39, 40], but the mechanisms remain unclear.

There are also questions regarding how human IgG reaches the brain parenchyma to cause these remarkably long-term effects. Consistent with our previous data [15], human IgG was found to transfer from dam to fetus throughout the last 6 days of gestation and to achieve levels of IgG and CASPR2-antibodies that were easily detectable in the embryos, and in the mice culled shortly after birth. Although CASPR2-antibodies were not at very high titer, the levels were similar to those we found in maternal samples [8]. Moreover, since transport in rodents is predominantly via lactation [2], in Experiment 3, lactation could have contributed to CASPR2-antibody exposure, while the levels remained high in the dams. The evidence of diffusion of IgG from the embryonic vasculature into the brain tissue that we found, despite the presence of a blood–brain barrier in the developing mouse [29], is similar to that shown using monoclonal antibodies to NR2a/2b subunits of the NMDA receptor in an active immunization protocol [16], and it is possible that the bound IgG may be sequestered in the parenchyma as recently proposed in humans and adult animals [6].

There are some limitations to this study. First, due to the lack of availability of maternal samples and need for large volumes of the injected material, we used IgG from two patients with CASPR2 encephalitis rather than IgG from mothers of children with neurodevelopmental disorders; it is possible that the CASPR2-antibodies in the two patients target different epitopes to those found in the mothers’ samples we previously examined [8]. Second, although we excluded the presence of other antibodies by binding to CASPR2-null brain tissue, it is still possible that low levels of other brain-reactive antibodies could have been present, but undetected, in the IgG preparations. Nevertheless, the phenotype similarities between our model and that using a CASPR2 monoclonal antibody derived from the mother of one child with autism [4] support our conclusion that the effects of the IgG preparations were due to the presence of the CASPR2-antibodies rather than other brain-reactive IgGs. Third, in a maternal-to-fetal transfer model, both dam and embryo are exposed to the pathogenic antibodies; it is possible that an effect of the human IgG on the dams’ maternal behavior could have influenced the development of the offspring. However, the adult blood–brain barrier is, under normal circumstances, only very slightly permeable to IgG, whereas others have shown [3] as we have here, that IgG can be found in the fetal brain parenchyma after transfer from the pregnant dam. It is possible that there were minor effects of the injected CASPR2-IgG on the maternal cognition or behavior, but this was not evident from observation of the dams themselves, nor in the lack of differences between the groups in neonatal milestones or physical maturation readouts, or in the maternal retrieval test.

Despite these limitations, our findings support a model in which maternal antibodies towards fetal cell-surface neuronal proteins can cause long-term behavioral deficits and permanent abnormalities at the cellular and synaptic levels in a small subset of children with neurodevelopmental disorders. The exact pathophysiological mechanisms by which CASPR2-antibodies affect brain development during the fetal period need further study, and how these result in such striking and persistent microglial and synaptic changes. These questions and the search for other maternal neuronal-surface antibodies indicate just some of the many avenues for further studies. Importantly, the identification of specific pathogenic antibodies in mothers of children with neurodevelopmental disorders could allow the identification of at-risk children, facilitating diagnosis and early intervention, and could also lead to treatment or preventive measures during the pregnancy, in keeping with the current practice in other antibody-mediated neonatal disorders.

## Electronic supplementary material

Below is the link to the electronic supplementary material.

Supplementary material 1 (PDF 2242 kb)
