# Supplementary material for: Persistent microglial activation and synaptic loss with behavioral abnormalities in mouse offspring exposed to CASPR2-antibodies in utero
Source: Acta Neuropathol. 2017 Jul 28;134(4):567–83. doi: 10.1007/s00401-017-1751-5 (PMC5587616; doi:10.1007/s00401-017-1751-5)
Supplement: Supplementary file 1 — Supplementary material 1 (PDF 2242 kb) [file 401_2017_1751_MOESM1_ESM.pdf]

Persistent microglial activation and synaptic loss with behavioral abnormalities in mouse offspring exposed to CASPR2-antibodies *in utero.*

Acta Neuropathologica

Ester Coutinho[1], David A. Menassa[1], Leslie Jacobson[1], Steven J. West[1], Joana Domingos[1], Teresa Moloney[1], Bethan Lang[1], Paul J Harrison[2,3], David LH. Bennett[1], David Bannerman[4]*, Angela Vincent[1]*

Affiliations: 1 -Nuffield Department of Clinical Neurosciences, University of Oxford, UK; 2- Department of Psychiatry, University of Oxford, UK; 3 - Oxford Health NHS Foundation Trust, Oxford, UK; 4 - Department of Experimental Psychology, University of Oxford, UK

Corresponding author: angela.vincent@ndcn.ox.ac.uk

Contents:

Online Resource 1: Patients' clinical information and plasma antibody titers.

Online Resource 2: Number of injected dams and injected material description for each experiment.

Online Resource 3: Supplementary material

Online Resource 4: Embryonic offspring (E18.5) global and brain morphology.

Online Resource 5: Total CASPR2 expression in CASPR2-IgG and HC-IgG exposed embryos at E18.5

Online Resource 6: 3-chamber social interaction test (raw data)

Online Resource 7: Locomotor activity during the habituation period of the social interaction tests

Online Resource 8: Gender effects on behavioral parameters

Online Resource 9: Morphometric analysis of CASPR2- and HC-IgG exposed offspring brain regions

Online Resource 10: Histological characterization of the corpus callosum

Online Resource 11: Histological characterization of the cerebellum

Online Resource 12: Histological analysis of the hippocampus

Online Resource 13: Histological analysis of the prefrontal cortex

Online Resource 14: Reactive microglia densities in the embryonic brain at E18.5

Online Resource 15: Staining for synaptic markers and quantification of PSD-95 synaptic profiles

Online Resource 16: Total GluA1 expression in CASPR2-IgG and HC-IgG exposed embryos at E18.5

Online Resource 1. Patients' clinical information and plasma antibody titers

Patient	Clinical information	Plasma CASPR2 CBA results
CASPR2 (1)	Male. Morvan's syndrome secondary to high VGKC antibodies / CASPR2 antibodies. Paraneoplastic cause excluded. At the age of 70, presented with cerebellar ataxia and progressive, disabling neuropathic pain in his feet and hands, memory complaints (short and long term memory, and particularly word-finding difficulties) and short episodes of lack of perception with "goosebumps" suggestive of temporal seizures. Partial response to plasma exchange was achieved when the CASPR2 antibody was identified (2 years after disease onset) and immunosuppressive therapy.	CASPR2 CBA titer: 1:4000
CASPR2 (2)	Male. Morvan's syndrome secondary to high VGKC antibodies / CASPR2 antibodies. At the age of 76, symptoms suggestive of neuromyotonia, excessive sweating and salivation, small joint pain and weight loss. Over 12 months, there was progressive deterioration with sleep disturbance, confusion and hallucinations. 10 plasma exchange courses were initiated 15 months after disease onset, with marked improvement of sleep and neuropsychiatric symptoms but over the next month there was progressive worsening of insomnia and diurnal drowsiness that disappeared after a new cycle of plasma exchange. Two-months later the patient suffered a new relapse and died during the further course of plasma exchange cycle. A pulmonary adenocarcinoma was detected postmortem (relevance to CASPR2 antibody unclear). Plasma collected during the last course of plasma exchange was used for IgG purification. Full detailed report in Liguori, Vincent et al. Brain 2001.	CASPR2 CBA titer: 1:8000
HC (1-3)	HC 1 – Male, 71 years old; HC 1- Male, 73 years old; HC 3 – Male, 40 years old	CASPR2 CBA: negative (<1:20)

Online Resource 2. Number of injected dams and injected material description for each experiment

	Patients		Controls			Antibody titer in pooled pups' sera
	CASPR2 (1)	CASPR2 (2)	HC (1)	HC (2)	HC (3)	
Experiment 1						
Injected dams	4					1:40*
IgG (mg/mL)<br>CBA titer	15<br>1:6400					
Experiment 2						
Injected dams	3		1	1	1	1:200**
IgG (mg/mL)<br>CBA titer	20<br>1:12500		20<br>neg	20<br>neg	20<br>neg	
Experiment 3						
Injected dams	2	2	2	2		1:100-1:200***
IgG (mg/mL)<br>CBA titer	20<br>1:12500	20<br>1:27000	20<br>neg	20<br>neg		

* At P0; ** At E18.5; ***At P1

Online Resource 3. Supplementary material

Immunological

Human IgG quantification in mice sera (experiment 1)

The levels of total human IgG in mice sera (dams and fetuses/pups) from experiment 1 were determined by quantitative western blotting. Human IgG standards of known concentration (BINDARID human IgG NL RID kit; Binding site) and mouse sera from the different gestational time points were diluted in deionized water. The diluted samples and standards were mixed with appropriate amounts of NuPAGE sample reducing agent (10x; Invitrogen, NP0009) and LDS sample buffer (4x; Invitrogen,) and the mixed solution was boiled for 5 minutes at 90ºC. 10 µL of the mixed sera and calibrators and 10µL of SeeBlue Plus2 pre-stained standard molecular weight ladder (Life Technologies, LC5925) were loaded into a 4-12% NuPAGE Bis-Tris SDS polyacrylamine gel (Invitrogen, NP0322). The gel was placed in a gel electrophoresis tank (XCell Sure Lock gel tank, Invitrogen) containing 1x MES SDS Running buffer (NuPage, Invitrogen, NP0002) and electrophoresed at 150mV for 1-1.5 hours. After electrophoresis, gels were removed and assembled in an X Cell II Blotting Module (Invitrogen, UK) and proteins transferred at 50V for 90 minutes in a 10% methanol transfer buffer (NuPage, Invitrogen, NP0006-1) into a nitrocellulose membrane. The membrane was then blocked in 5% BSA in PBS/0.1% Tween for 30 minutes and then incubated with rabbit anti-human IgG HRP (Dako, P0214; 1:2000) for 1 hour at room temperature. Next, the blot was washed 3 times in PBS/0.1% tween and incubated with ECL (Pierce, 32106) for 2 minutes. The membrane was exposed to photographic film in the dark until signal was visualized. The photographic film was then scanned and analysed with Image J. Mean gray values for each band were measured and the optical density calculated. To estimate the specific protein density, the optical density readings were corrected for non-specific background density, measured in an area of the blot with no reactivity. A calibration curve was then created on Graphpad software (San Diego, California, USA) using the specific optical density readings of the known standards and the mice sera samples' concentration inferred.

Antibody titers in mice sera (experiment 1-3)

Dams and fetus/pups sera from all experiments were diluted from 1:5 to 1:400 and tested for specific antibodies. Antibody titers were established by end-point titration on a cell based assay, as previously described (Irani *et al.,* 2010).

Histology of embryonic tissue (experiment 2)

Cresyl violet staining for visualization of the Nissl substance. Tissue sections were lightly stained with a cresyl violet solution (Sigma C5042) that stains Nissl bodies (granular endoplasmic reticulum and ribosomes) and allows observation of neuronal soma. After allowing the sections to dry at room temperature, they were fixed with 4% paraformaldehyde for 10 minutes, rinsed twice in PBS for 5 minutes and once in deionized water, followed by immersion in cresyl violet solution for 10 minutes. Slides were dehydrated in graded alcohols, cleared with xylene and coversliped with DPX mounting medium. Photomicrographs were scanned with the Aperio AT2 slide scanner and analyzed with the e-pathology Aperio Imagescope image analysis system from Leica Biosystems.

Elution of human IgG from embryonic tissues

The heads of 3 CASPR2-IgG and 3 HC-IgG exposed E18.5 embryos were homogenized in 200 µL/100mg tissue of PBS with protease inhibitors (1:100; SIGMA). The homogenate was centrifuged at 16.000g for five minutes at 4ºC. The supernatant was discarded and the pellet washed four times in ice-cold PBS. Sodium-citrate buffer (0.1M, pH 2.7), 100 µl, was added to the pellet and mixed for 5 minutes. After centrifugation at 16.000g for 5 minutes at 4ºC, the supernatant was collected and neutralized with 25µL of Tris-buffer (1.5M, pH 8.8). The elution was then tested in a live CASPR2 cell-based assay, as described above.

Behavioral testing (experiment 3)

At P1, 6 pups were randomly selected from each litter and individually tattooed, in order to provide a form of identification that was persistent throughout the entire period of behavioral testing. While the pups were tattooed, the dam was left in the home cage and placed in the testing room for habituation. The remaining pups from that litter were culled and blood collected for antibody testing, as previously described

Neonatal period.

Testing was done daily, from P1 to P21, in the dark phase (lights off at 7pm). Pups were separated from the dam and placed in a petri dish with home cage nesting material at the beginning of testing. All testing was done with the pups placed in a heating pad and, for the first five postnatal days, under a lamp with a 60 watt bulb. Pups were returned to the dam after testing. Care was taken to extensively rub the examiners hands and the pups in the home cage shavings in order to avoid maternal rejection. All material was disposed of or washed with tepid water between litters.

Modified Fox battery. Tests were performed by the same examiner daily and a second examiner would record the time of performance with a stopwatch. Weight was measured daily, from P1 to P21, for each individual pup, prior to the testing. If a pup did not reach criterion by the end of the specified testing days, the day following the last test day was used for statistical analysis.

Eye opening and pinna detachment. The mouse pup was examined daily, from P1 to P21, to determine the first day when both eyes were opened or both ears detached from the skull.

Surface righting. The mouse pup was held on its back, supported on both sides of the head and on the hind quarters, and released onto a smooth surface. The time for the pup to flip to its abdomen and touch the surface with all four paws was measured in seconds. If the pup could not reach that position within 30 seconds, the test was terminated. The test was performed from P1 to P13 or until the pup could reach that position in less than 1 second for two consecutive days

Negative geotaxis. The pup was placed in a screen at a 45º angle with its head facing down. The time for the pup to turn 180º, so the head faced up, was measured in seconds. If the pup fell, the test was repeated once. If the pup could not reach that position within 30 seconds, the test was terminated. The test was performed from P1 to P14 or until the pup could reach that position in less than 30 seconds for two consecutive days.

Cliff aversion. The mouse pup was positioned on the top of a box with the forepaws and snout placed on the edge of the box. Time for the pup to crawl away from the edge of the box was measured in seconds. If the pup fell, the test was repeated once. If the pup could not reach that position within 30 seconds, the test was terminated. The test was performed from P1 to P14 or until the pup could reach that position in less than 30 seconds for two consecutive days.

Rooting. A cotton filament was used to stroke three times the pup's head, laterally, from front to back. A rooting response was considered to occur if the pup moved the head towards the filament. If no response occurred, the other side of the head was stroked in the same way. The test was performed from P1 to P12 or until a response was elicited for two consecutive days.

Ear twitch. A cotton filament was used to stroke three times the pup's ear tip. A response was considered to occur if the mouse would flatten the ear against the side of the head. If no response occurred, the other ear was stroked in the same way. The test was performed from P7 to P15 or until a response was elicited for two consecutive days.

Air righting. The mouse pup was held on its back, supported on both sides of the head and on the hind quarters, and released approximately 10cm above a clean cage padded with nesting material. The day in which the pup would flip to its abdomen and land on the surface with all four paws was recorded. The test was performed from P8 to P21 or until the pup could reach that position for two consecutive days.

Forelimb grasping. The pup was held supported on both sides of its trunk and the forepaws placed on a small rod (approximately 5mm in diameter) suspended over a clean cage padded with nesting material. The pup was then released and the amount of time the mouse remained gripping the rod was measured. If the mouse fell immediately after release, the test was repeated once. The test was performed from P4 to P14 or until the mouse could hold the grip for 1 second or more for two consecutive days.

Open field traversal. The pup was placed in the center of a circle with a 13cm diameter printed on a sheet of paper. The amount of time the mouse would take to move outside the circle with all four paws was recorded. If the mouse failed to leave the circle in 30 seconds, the test was terminated. The test was performed from P8 to P21 or until the mouse would reach criterion in less than 30 seconds in two consecutive days.

Maternal retrieval test. From each litter, 3 pups were isolated from the dam and littermates for 2 minutes. After this period, the dam was isolated in a clean cage and the 3 pups returned to the home cage approximately 20 cm from the nest. The dam was then returned to the box, at the opposite corner from the 3 pups, facing away. Time in seconds was recorded until retrieval of the first pup (latency to retrieval) and until retrieval of the 3 pups to the nest (time to retrieval). Total test duration was 5 minutes. If the dam failed to retrieve the 3 pups at the end of this period, 300 seconds was used for statistical purposes. Test was performed at P6.

Adult period

The pups were weaned from the dam at P21 and separated according to sex. To avoid isolation, if a litter only had one male or one female, that animal would be placed with a different litter. Animals were housed in groups of two to five. Behavioral testing was done from 4 to 10 months of age during the light phase. Unless indicated otherwise, behavior tests were performed in half of the cohort (12 animals per treatment group, 6 males and 6 females), randomly selected.

Locomotor activity. To assess locomotor activity, mice were transferred to a novel cage (42cm long x 22cm wide x 20cm high) placed within a photobeam frame (San Diego instruments, San Diego, CA, USA) and activity was quantified as number of beam breaks during a 2-hour session. Data was divided in 24 five minutes bins and analyzed. This test was done in all mice (except 1 HC IgG-exposed mouse, deceased due to a non-experimental reason).

Accelerating rotarod. Neuromotor coordination was assessed using the accelerating rotarod (Deacon, 2013). The animals were brought to the experimental room approximately 15 minutes before testing. The mouse was placed using a dowel on the rotating rod at an angle of 30

degrees (head down), facing away from the direction of rotation. Steady starting speed of 4 rotations per minute was maintained during 10 seconds. Afterwards, an acceleration rate of 20 rotations per minute ensued. Time to fall was recorded. If the mouse had fallen off during the initial 10 seconds it would have another try, to a maximum of 3 trials.

Elevated Plus-maze (EPM). Anxiety was assessed using the EPM. The apparatus is in the shape of a plus sign, made up of two opposite 27 x 8 cm x 30 cm enclosed black arms, a 12.5 cm x 6 cm black central rectangular area and two opposite 29 x 4 x 0.5 cm open white arms, and is elevated 50 cm from the floor on a stand. Mice were brought to the experimental room approximately 15 minutes before testing. The mouse was placed at the central rectangular area facing the open arms. Behavior was then video recorded for 5 min and analyzed using the Ethovision software. Recorded parameters were: total distance travelled, latencies to enter four paws into an open and closed arm, total time spent in enclosed and open arms and number of entries into enclosed and open arms.

Light-dark (LD) box. The LD box test was also used to assess anxiety. The apparatus consists of an open white compartment 30 x 20 x 20 cm joined by a 3 x 3 cm opening to a dark box 15 x 20 x 20 cm. The white side of the box was transparent allowing detection of the mouse movement and was further illuminated by a 60W lamp. The mouse was placed in the middle of the dark side facing away from the opening and a count-down timer for 5 min started. The latency to cross with all four feet to the light side, the total time spent on the light side and the number of transitions through the opening were registered for the test duration.

T-maze spontaneous alternation test. The T maze spontaneous alternation test is based on the natural tendency of rodents to alternate their choice arm, as a manifestation of the animal’s motivation to explore its environment (Deacon and Rawlins 2006). The T-maze consists of a T-shaped apparatus (30cm long x 10 cm wide x 29 cm high) with one start arm and 2 goal arms. It further has a removable central partition extending 7 cm into the start arm from the back of the T, restricting access to only one goal arm at a time and three guillotine doors that can restrict animal to either goal arm or the first 22.5 cm of the start arm. The floor of the apparatus was covered with a layer of soiled bedding from cages holding animals unknown to the test mouse, providing a stimulus for exploration. The bedding was mixed between animals and changed between trials. The test consists of a sampling and a choice phase. During the sample phase, the animal was placed at the end of the start arm facing away from goal arms. During this phase, the central partition was in place. Once the mouse entered a goal arm, the guillotine door on that side was gently closed and the animal was allowed to explore for 30 seconds. The animal was then removed from the goal arm, the central partition removed and the guillotine doors opened, allowing free choice of goal arm for second exploration. The mouse was then returned to the start arm and the choice recorded. Latency to choice was also recorded. The test was run in pairs of trials, once in the morning and once in the afternoon for 5 consecutive days. This test was done in all mice (except for the above mentioned HC IgG-exposed mouse).

Three-chamber social interaction test. The three-chamber social interaction test is an automated method to measure non-reciprocal social interaction (Nadler *et al.*, 2004). The test has 3 phases: a habituation period, a sociability phase (when the test mouse chooses to interact with an empty cage or a stimulus mouse) and a social memory phase (when the test mouse chooses to interact with the known stimulus mouse or with an unknown stimulus mouse).

The three-chamber apparatus is a rectangular Plexiglas box (60 x 30cm for whole arena). The whole arena is divided into three equal parts by red-prespex slider walls (each chamber is 30 x

20cm; walls are 15cm high), that control the access to the lateral chambers. Stimulus mice (gender-matched CD1 outbreds) were extensively habituated to the enclosure (a wire cage) previously to the test. Stimulus mice were separated into 2 groups and housed in different cages in order to have distinct smells. Habituation protocol for the stimulus mice included: free exploration of the apparatus (same-cage animals would explore the chamber at the same time) for five minutes, enclosure of a single mouse during free exploration (first session for 40 seconds; second session for 1 minute; each stimulus mouse enclosed for 3 times in each session), direct enclosure of a single mouse (first session for 2 minutes, second session for 10 minutes; each stimulus mouse enclosed 2 times in each session), and direct enclosure of the stimulus mouse with a second mouse (from a different group) freely-exploring for 5 minutes. Each stimulus mouse was used 2 times per test day, at maximum. In the test session, a test mouse would be placed in the middle chamber and allowed to explore for 5 minutes. Afterwards, the slide doors would be opened and the mouse would explore the entire empty chamber for 10 minutes. The mouse would then be enclosed in the middle chamber, briefly, while an empty cage and a cage with a novel stimulus mouse were placed in opposite sides of the apparatus. The test mouse would then be released, by opening of the sliding doors, and allowed to explore the entire apparatus. The object and novel mouse position alternated across subjects. Next, the test mouse was again enclosed in the central chamber, and the empty enclosure replaced by an enclosure with a new stimulus mouse (from a different group). The test mouse was again released and allowed to explore the apparatus for 10 minutes. The novel and old mouse position alternated across subjects. The test session was video recorded and analyzed by the Any-Maze tracking software (Stoelting, USA). Parameters analyzed included: distance travelled, freezing and immobility times in the habituation phases, and in the sociability and social memory phases, absolute time in interaction zone (2cm area around enclosure), time spent in each chamber, number of entries into interaction zone. The test was run in the dark, with two desk lamps facing away from the arena on each side and using red-light; background noise was masked with a white noise amplifier. The apparatus and enclosures were cleaned with 70% alcohol and water between subjects.

Reciprocal social interaction test. In the reciprocal social interaction test two mice of the same treatment group, unknown to each other, were allowed to interact freely (Barkus, 2012). The apparatus consisted of a clean cage (41.5 × 25.5 ×11.5 cm) with bedding sufficient to cover the bottom of the cage. Each mouse was individually habituated to the testing arena on 5 consecutive days, 10 minutes per day. Habituation sessions were video-recorded and analyzed with the Any-maze software (distance travelled, freezing and immobility times) to ensure equal exploratory activity between animals. On the sixth day, each mouse was exposed for 10 minutes to a mouse from the same treatment group but housed in a different cage. The animals were sex-matched and tightly matched for weight (within 5% difference). This was repeated on the seventh day with new pairs. No animal was tested more than twice. The test was video recorded and scored offline for time spent in social and non-social behaviors and number of social and non-social events. Social behaviors included sniffing, grooming and following closely; non-social behaviors included self-grooming and digging. Testing room conditions were the same as for the three-chamber social interaction test. The apparatus was cleaned between tests with 70% ethanol and water.

Nesting test. Approximately 1 hour before the dark phase, the animals were transferred to individual cages with wood-chip bedding and without any other environmental enrichment. A cotton nestlet weighing 2.5 grams was placed in each cage. The following morning, the nests were scored according to (Deacon, 2006).

Olfaction test. The apparatus from the three chamber social interaction test was used in order to benefit from previous habituation. A small glass container with an odor (1 mL of banana or peppermint food flavoring) was placed at each corner. The test comprises 3 phases: a sample phase (15 minutes), a delay period (15 minutes) and a test phase (3 minutes). During the first phase, the same odor is placed in both containers (half of the mice for treatment group would smell one odor, while the other half would smell the other). After this, the mice would return to the home cage for 15 minutes. During the test phase, a container with the alternate (new) odor was placed in one of the corners. The location of the new odor was counterbalanced across treatment groups and gender. Testing room conditions were the same as for the social interaction tests. The test was video recorded and analyzed using the Any-maze software. The same parameters as in the 3-chamber social interaction test were analyzed.

Histological analysis of adult offspring (experiment 3)

Morphological and morphometric analysis. Morphology and morphometry were assessed in one series of Nissl stained sections. Gross morphology of the entire brain and of specific regions (cortex, hippocampus, corpus callosum and cerebellum) was assessed. Sections were scanned using the Aperio AT2-scanner and analyzed with the e-pathology Aperio Imagescope image analysis system from Leica Biosystems, which allowed for thickness and area measurements.

Neuronal and astrocytic immunohistochemistry. Neurons and astrocytes were identified by immunofluorescence using a polyclonal rabbit anti-glial fibrillary acidic protein (GFAP) antibody (Dako, Z0334; 1:500) and mouse anti-NeuN (Chemicon, MAB377; 1:500) in free-floating sections. Sections were fixed with 4% formaldehyde, washed with PBS then blocked with 10% normal goat serum (Vector laboratories, S-1000) in PBS-Triton-X-100 (0.3%) for an hour then incubated overnight with primary antibodies at 4ºC. The sections were washed the next day with PBS then incubated for two hours at room temperature with goat anti-rabbit (568) and goat anti-mouse (488) Alexa-fluor secondary antibodies from life technologies at 1:1000 dilutions in the dark. Sections were subsequently washed in PBS, mounted on slides after a brief TNS wash (pH = 7.4) and counterstained with DAPI mounting medium from Vector Laboratories (UK), left to dry for an hour then sealed and stored protected from the light at 4ºC for confocal imaging.

Cortical layers immunohistochemistry. Deep layers (layers V and VI) of the neocortex were identified by immunofluorescence using a goat anti-FOXP2 antibody (SantaCruz (sc-21069); 1:50) and the middle layers (layers II/III) using a rabbit anti-CUX-1 antibody (SantaCruz (sc-13024); 1:200) in free-floating sections, following the previously described laminar-specific genes in the mouse neocortex (Molyneaux, Arlotta et al. 2007). Sections were fixed with 4% formaldehyde, washed with PBS then blocked with 10% normal donkey serum in PBS-Triton-X-100 (0.3%) for an hour then incubated for 48 hours with primary antibodies at 4ºC. The sections were washed the next day with PBS then incubated for three hours at room temperature with donkey anti-rabbit (568) and donkey anti-goat (488) Alexa-fluor secondary antibodies from life technologies at 1:500 dilutions and kept in the dark. Sections were subsequently washed in PBS, mounted on slides after a brief TNS wash (pH = 7.4) and counterstained with DAPI mounting medium from Vector Laboratories (UK), left to dry for an hour then sealed and stored protected from the light at 4ºC for confocal imaging.

Activated microglia immunohistochemistry. Reactive microglial cells were identified by immunofluorescence using a rat anti-CD68 antibody (BioRad, MCA1957; 1:400) and a rabbit anti-Iba1 antibody (Wako chemicals, 019-19741) in free-floating sections. Sections were fixed with 4% formaldehyde, washed with PBS then blocked with 10% normal donkey serum

in PBS-Triton-X-100 (0.3%) for an hour then incubated for 48 hours with primary antibodies at 4ºC. The sections were washed the next day with PBS then incubated for three hours at room temperature with goat anti-rat (488) Alexa-fluor secondary antibody from life technologies at 1:500 dilution and kept in the dark. Sections were subsequently washed in PBS, mounted on slides after a brief TNS wash (pH = 7.4) and counterstained with DAPI mounting medium from Vector Laboratories (UK), left to dry for an hour then sealed and stored protected from the light at 4ºC for confocal imaging.

Microglia morphology macro:

```
run("Z Project...", "projection=[Max Intensity]");
run("Duplicate...", "duplicate channels=3");
setAutoThreshold("Huang");
setOption("BlackBackground", false);
run("Convert to Mask");
run("Despeckle");
run("Invert");
run("Analyze Particles...", "size=500-Infinity pixel show=Masks clear");
```

References

Barkus C, Feyder M, Graybeal C, Wright T, Wiedholz L, Izquierdo A, *et al*. "Do GluA1 knockout mice exhibit behavioral abnormalities relevant to the negative or cognitive symptoms of schizophrenia and schizoaffective disorder?" Neuropharmacology 2012; 62(3): 1263-1272.

Deacon RM. "Assessing nest building in mice." Nat Protoc 2006; 1(3): 1117-1119.

Deacon RM, Rawlins JN. "T-maze alternation in the rodent." Nat Protoc 2006; 1(1): 7-12.

Deacon RM. "Measuring motor coordination in mice." J Vis Exp 2013; (75): e2609.

Irani SR, Alexander S, Waters P, Kleopa KA, Pettingill P, Zuliani L, *et al*. "Antibodies to Kv1 potassium channel-complex proteins leucine-rich, glioma inactivated 1 protein and contactin-associated protein-2 in limbic encephalitis, Morvan's syndrome and acquired neuromyotonia." Brain 2010; 133(9): 2734-2748.

Nadler JJ, Moy SS, Dold G, Trang D, Simmons N, Perez A, *et al*. "Automated apparatus for quantitation of social approach behaviors in mice." Genes Brain Behav 2004; 3(5): 303-314.

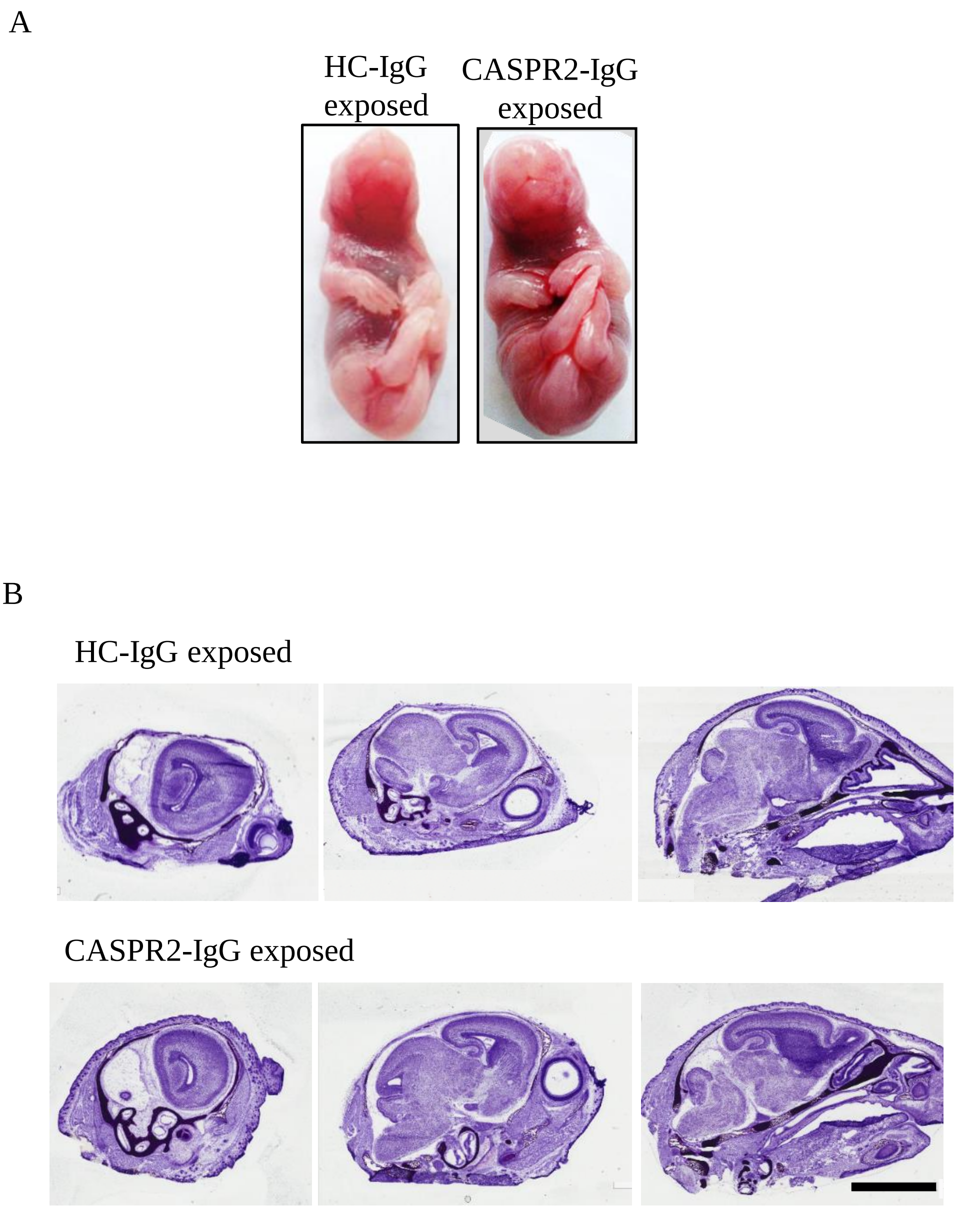

Online Resource 4. Embryonic offspring (E18.5) global and brain morphology.

(A) Representative photographs from E18.5 embryos from HC-IgG and CASPR2-IgG treatment groups. (B) Representative images of the Nissl staining performed in every 10th sagittal section for each animal. Scale bar: 3mm.

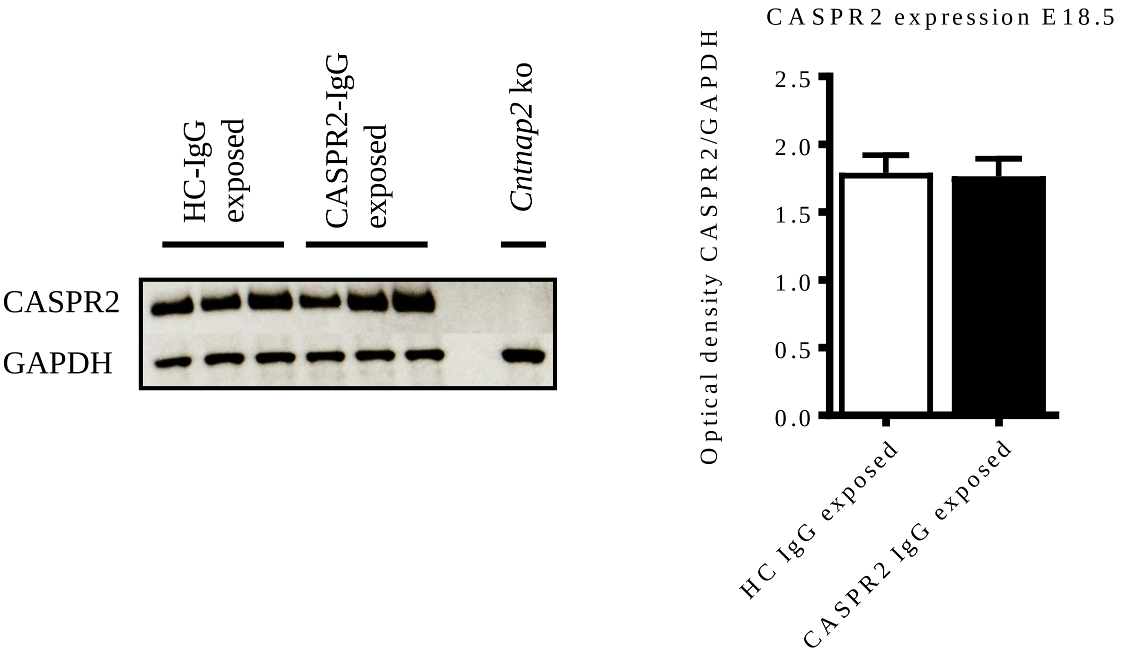

Online Resource 5. Total CASPR2 expression in CASPR2-IgG and HC-IgG exposed embryos at E18.5

Data presented as mean ± SEM of two western blot replicates from 3 mice/group.

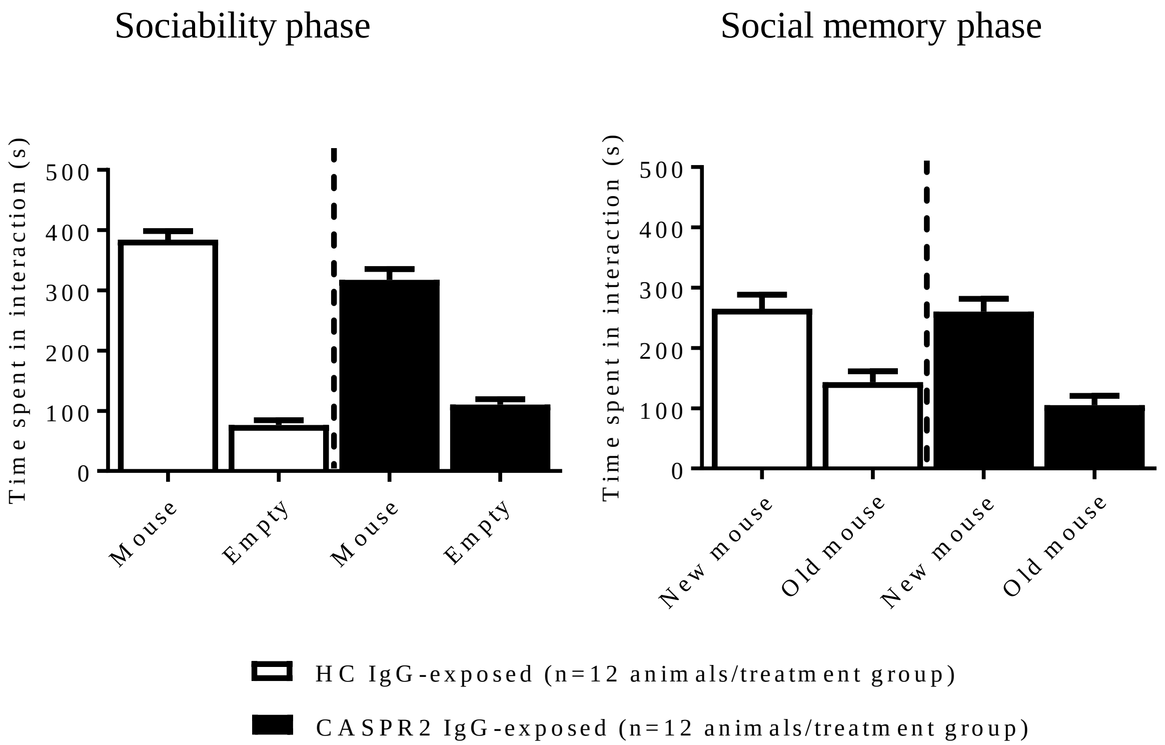

	HC-IgG exposed (Mean ± SEM)	CASPR2-IgG exposed (Mean ± SEM)
Sociability phase		
Time spent in interaction with mouse	384.1 (±13.9)	317.5 (±17.1)
Time spent in interaction with empty cage	76.4 (±7.8)	110.2 (±8.6)
Social memory phase		
Time spent in interaction with new mouse	265.3 (±22.1)	260.4 (±20.2)
Time spent in interaction with old mouse	143.2 (±17.1)	104.9 (14.9)

Online Resource 6. 3-chamber social interaction test (raw data).

A. Three-chambers social interaction test

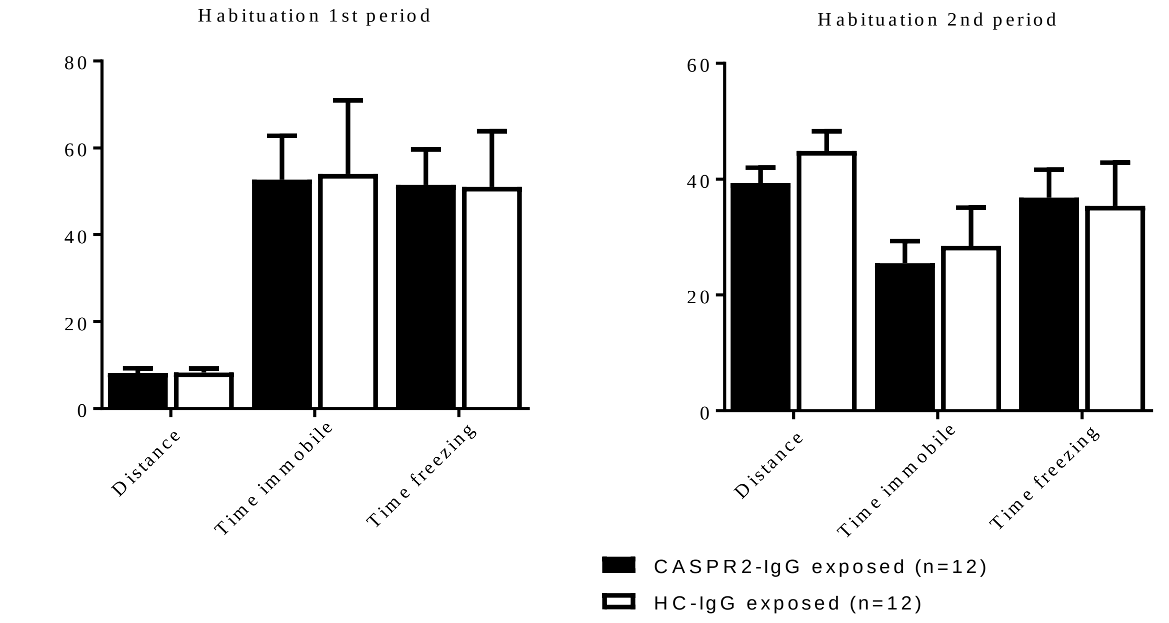

B. Reciprocal social interaction test

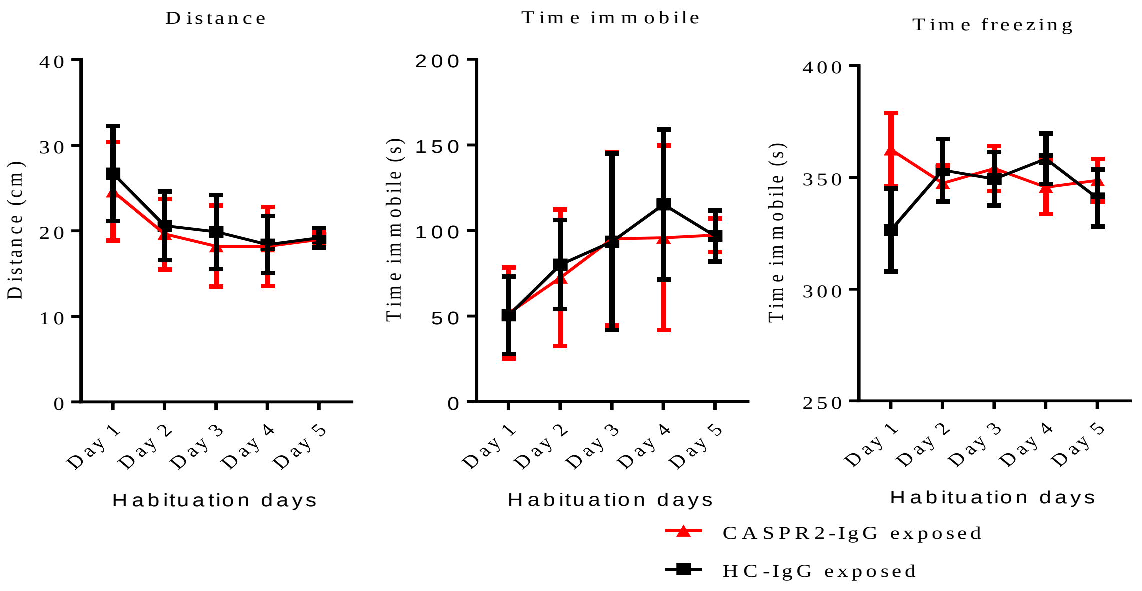

Online Resource 7. Locomotor activity during the habituation period of the social interaction tests

No statistically significant differences between groups in distance travelled, time immobile and time freezing during 1st and 2nd period of habituation in the three chambers social interaction test (A) or in the 5 days of habituation to the apparatus in the reciprocal interaction test (B), confirming that the social deficits observed are not due to differences in the exploratory behavior.

Online Resource 8. Gender effects on behavioral parameters

	CASPR2-IgG		HC-IgG		
	Males Mean (±SD)	Females Mean (±SD)	Males Mean (±SD)	Females Mean (±SD)	GENDER INTERACTION
Locomotor activity (mean nr beam breaks)	174.9 (±15.6), n=10	193.9 (±13.2), n=14	178 (±13.2), n=14	193.1 (±15.6) n=10	Time*Group*Gender: F(9.49,417.6)=1.59; *P*=0.11
Rotarod (mean time to fall)	54.3 (±7.5), n=6	47.2 (±7.5), n=6	51.6 (±7.5), n=6	56.1 (±7.5), n=6	Rot_Session*Group*Gender: F(1,20)=1.84; *P*=0.19
Light-dark box - time spent in the light	23.5 (±21.1), n=6	46.7 (±38.1), n=6	94.7 (±42.8), n=6	48.1 (±66.4), n=6	Group*Gender: F(1,20)=3.59; *P*=0.07
Light-dark box - latency to the light	125.0 (±115.0), n=6	102.8 (±95.0), n=6	92.3 (±67.8), n=6	198.8 (±116.2), n=6	Group*Gender: F(1,20)=2.46; *P*=0.13
Light-dark box - entries in the light	2.2 (±2.5), n=6	4.5 (±3.2), n=6	6.0 (±3.3), n=6	3.2 (±3.5), n=6	Group*Gender: F(1,20)=4.02; *P*=0.06
Elevated plus-maze - total distance	1158.8 (±535.4), n=6	1306.6 (±271.1), n=6	1372.4 (±527.8), n=6	1184.0 (±272.1), n=6	Group*Gender: F(1,20)=0.95; *P*=0.34
Elevated plus-maze - time in open arm	48.9 (±40.1), n=6	9.6 (±8.6), n=6	42.6 (±49.5), n=6	7.74 (±6.4) n=6	Group*Gender: F(1,20)=0.05; *P*=0.82
Elevated plus-maze - entries in open arm	11.7 (±8.1), n=6	6.8 (±4.1), n=6	12.5 (±9.0), n=6	6.0 (±4.6), n=6	Group*Gender: F(1,20)=0.09; *P*=0.77
T-maze spontaneous alternation	59.0 (±21.8), n=10	70 (±18.8), n=14	71.4 (±17.5), n=14	82.2 (±13.9), n=9	Group*Gender: F(1,43)=0.0; *P*=0.99
Nesting_score	3.5 (±0.6), n=6	4.3 (±0.5), n=6	4.7 (±0.5), n=6	4.3 (±0.5), n=6	**Kruskal-Wallis: H(3)=9.6; *P*=0.02**<br>**Males – U=33; *P*=0.01**<br>Females – U=18; p=1.0
3-chamber social interaction- % IA time mouse (/object)	2.9 (±1.2), n=6	3.5 (±1.8), n=6	5.7 (±3.3), n=6	6.0 (±2.2), n=6	Group*Gender: F(1,20)=0.01; *P*=0.93
3-chamber social interaction- % IA time new mouse (/old mouse)	3.2 (±0.9), n=6	1.6 (±0.6), n=6	1.9 (±0.5), n=6	2.6 (±2.0), n=6	Group*Gender: F(1,20)=3.74; *P*=0.07
Reciprocal interaction - time in social contacts	129.5 (±57.2), n=6	112.4 (±32.6), n=9	193.6 (±60) n=4	172 (±30.3), n=2	Group*Gender: F(1,17)=0.01; *P*=0.93
Reciprocal interaction - time in non-social contacts	223.0 (±122.1), n=6	140 (±77.1), n=9	39.0 (±22), n=4	141.5 (±136.5), n=2	Group*Gender: F(1,17)=4.0; *P*=0.06
Olfaction test - % time new smell (test phase)	59.9 (±14.7), n=4	75.8 (±21.2), n=4	65.5 (±23.7), n=4	64.3 (±22.6), n=4	Group*Gender: F(1,12)=0.17; *P*=0.69
Olfaction test - % entries new smell (test phase)	60.1 (±10.7), n=4	71.0 (±17.7), n=4	66.2 (±16.7), n=4	64.0 (±15.3), n=4	Group*Gender: F(1,12)=0.73; *P*=0.41

Online Resource 9. Morphometric analysis of CASPR2- and HC-IgG exposed offspring brain regions

	CASPR2-IgG exposed (mean ±SEM)	HC-IgG exposed (mean ±SEM)	*P* value*
Total brain volume	385.6 (±7.0)	380.8 (±8.2)	n.s.
Corpus callosum			
Volume	7.0 (±0.4)	7.3 (±0.3)	n.s.
Thickness	185.5(±13.2)	191.1 (±16.9)	n.s.
Hippocampus			
Volume	8.8 (±1.8)	7.4 (±2.4)	n.s.
Cerebellum			
Volume	56.0 (±2.1)	56.6 (±2.8)	n.s.
Prefrontal cortex			
Prelimbic thickness	941.1 (±9.9)	974.6 (±16.9)	n.s.
Infralimbic thickness	711.3 (±9.9)	729.5 (±7.7)	n.s.
Anterior cingulate thickness	1041.9 (±30.1)	1047.3 (±22.9)	n.s.
Somatosensory cortex			
Layer I thickness	85.3 (±4.3)	87.8 (±5.2)	n.s.
Layer II-IV thickness	425.6 (±12.3)	410.3 (±10.1)	n.s.
Layer V-VI thickness	608.8 (±11.0)	628.8 (±14.1)	n.s.
Overall	1119.6 (±22.9)	1127.0 (±26.3)	n.s.

All values mm (thicknesses) or mm^3 (volumes). *Student's t-test: non-significant (n.s.) if $p>0.05$; n=6 brains/group

(i)

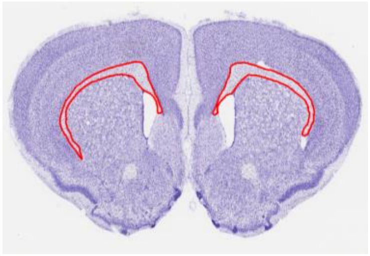

(ii)

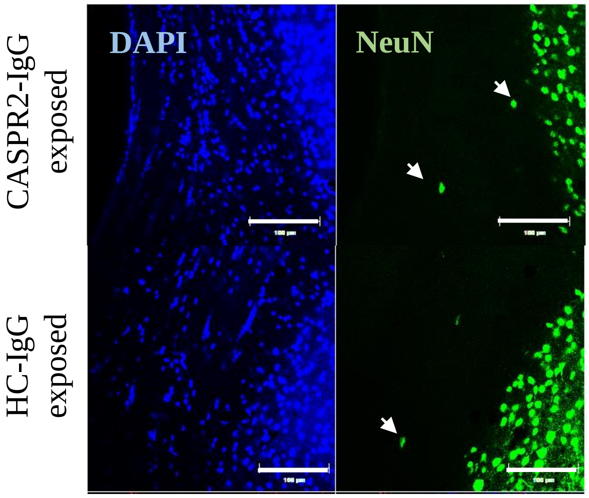

(iii)

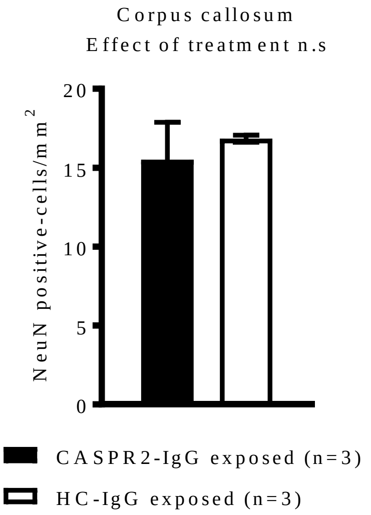

Online Resource 10. Histological characterization of the corpus callosum.

(i) Representative image of a Nissl-stained section showing the corpus callosum boundaries used for morphological and morphometric characterization. (ii) Representative images of the corpus callosum showing small number of ectopic neurons (arrows); DAPI, blue; Neuronal Nuclei (NeuN), green. Scale bar: 100 µm; (iii) Despite some variation, no differences were found between CASPR2- and HC-IgG in the neuronal densities in the corpus callosum (n=3 brains /treatment group). Data presented as mean±SEM.

(i)

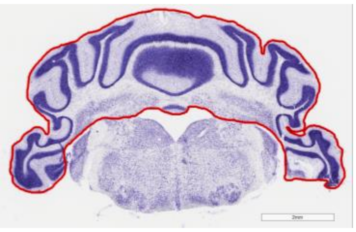

(ii)

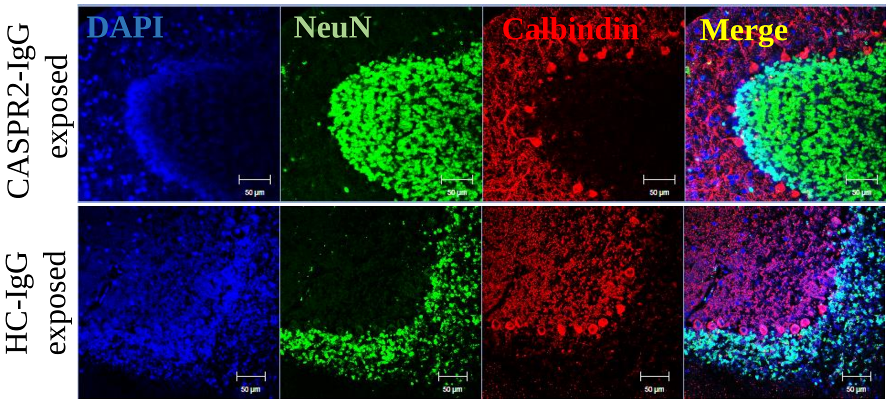

(iii)

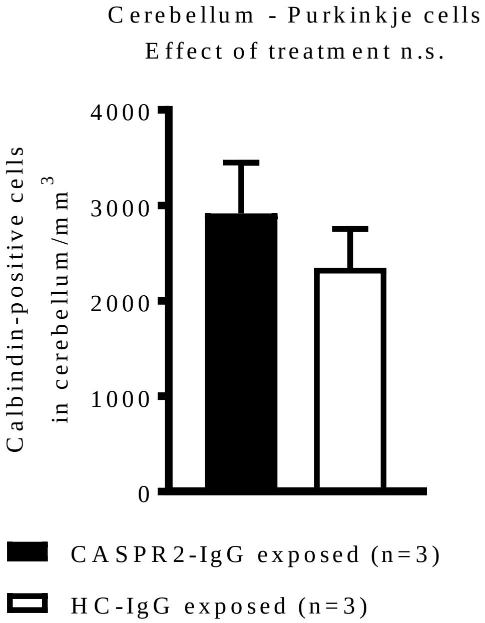

Online Resource 11. Histological characterization of the cerebellum.
(i) Representative image of a Nissl-stained section showing the cerebellum boundaries used for morphological and morphometric characterization. (ii) Representative images showing normal Purkinje cell line in all treatment groups (DAPI, blue; Neuronal Nuclei (NeuN), green; Calbindin, red). Scale bar: 50 µm; (iii) No differences found across treatment groups in the Purkinje cell densities (n=3 brains /group). Data presented as mean±SEM.

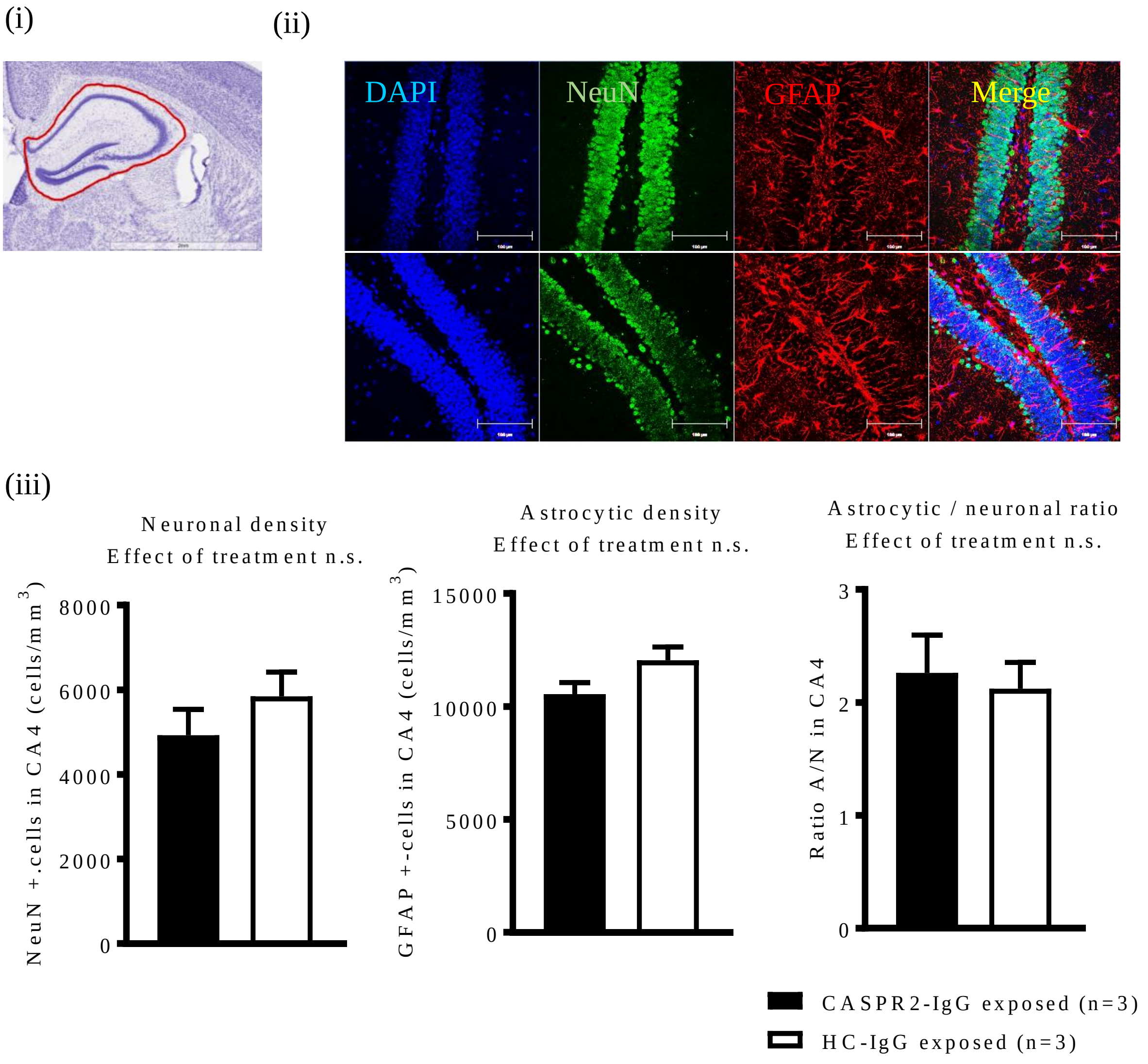

Online Resource 12. Histological analysis of the hippocampus.
(i) Representative image of a Nissl-stained section showing the hippocampus boundaries used for morphological and morphometric characterization. (ii) Representative images of the CA4 area of the hippocampus showing neuronal and astrocytic populations; DAPI, blue; Neuronal Nuclei (NeuN), green; Glial-fibrillary acidic protein (GFAP), red. Scale bar: 100 μm; (iii) No differences found across treatment groups in the neuronal density, astrocytic density or astrocytic/neuronal ratio in the CA4 area of the hippocampus (n=3 brains /group). Data presented as mean±SEM

(i)

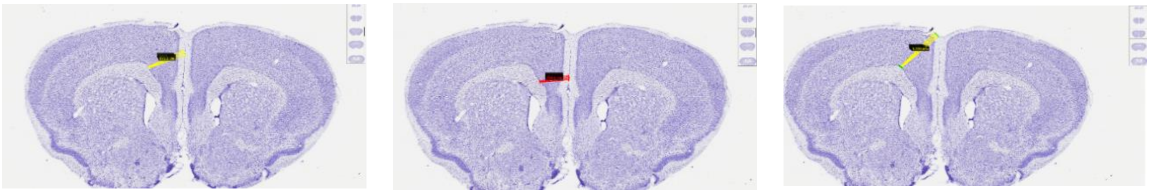

(ii)

CAS PR2-IgG exposed

HC-IgG exposed

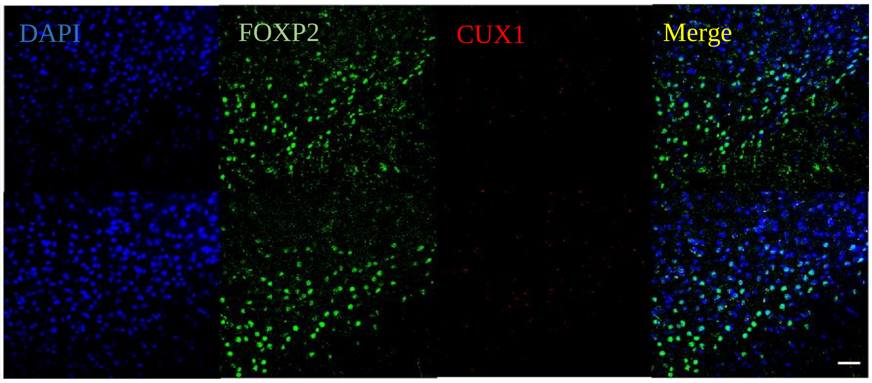

Online Resource 13. Histological analysis of the prefrontal cortex.
(i) Representative images of a Nissl-stained section showing the prelimbic, infralimbic and anterior cingulate cortex for morphological and morphometric characterization.
(ii) Representative images of cortical layering in the prelimbic cortex, focusing on layers V/VI. Scale bar: 50 μm

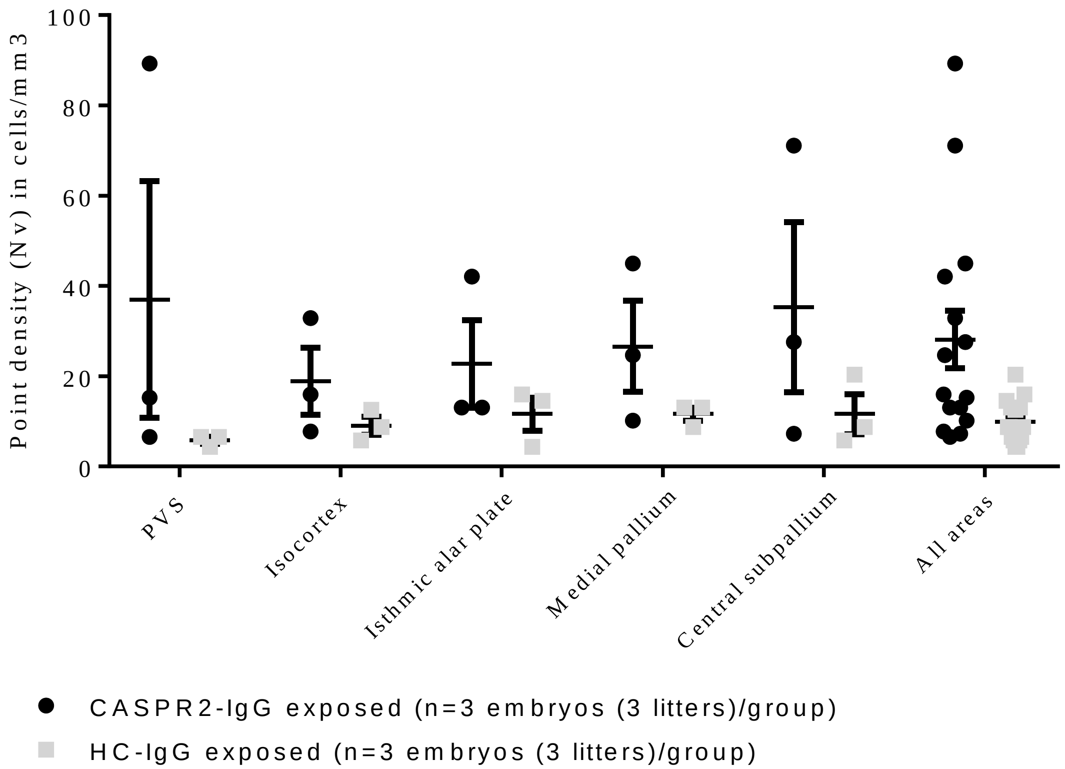

Online Resource 14. Reactive microglia densities in the embryonic brain at E18.5
No statistically significant differences found in microglial densities between CASPR2 and HC IgG-exposed embryos in the periventricular space (PVS; p=0.047); isocortex (p=0.52), isthmic alar plate (p=0.47), medial pallium (p=0.33), central subpallium (p=0.13), or in all combined observations (p=0.01); multiple t-test; uncorrected p-values. Data presented as mean±SEM.

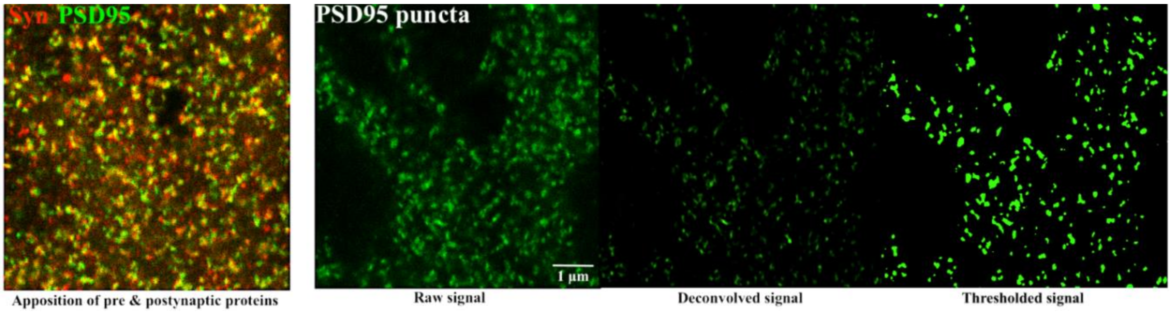

Online Resource 15. Staining for synaptic markers and quantification of PSD-95 synaptic profiles

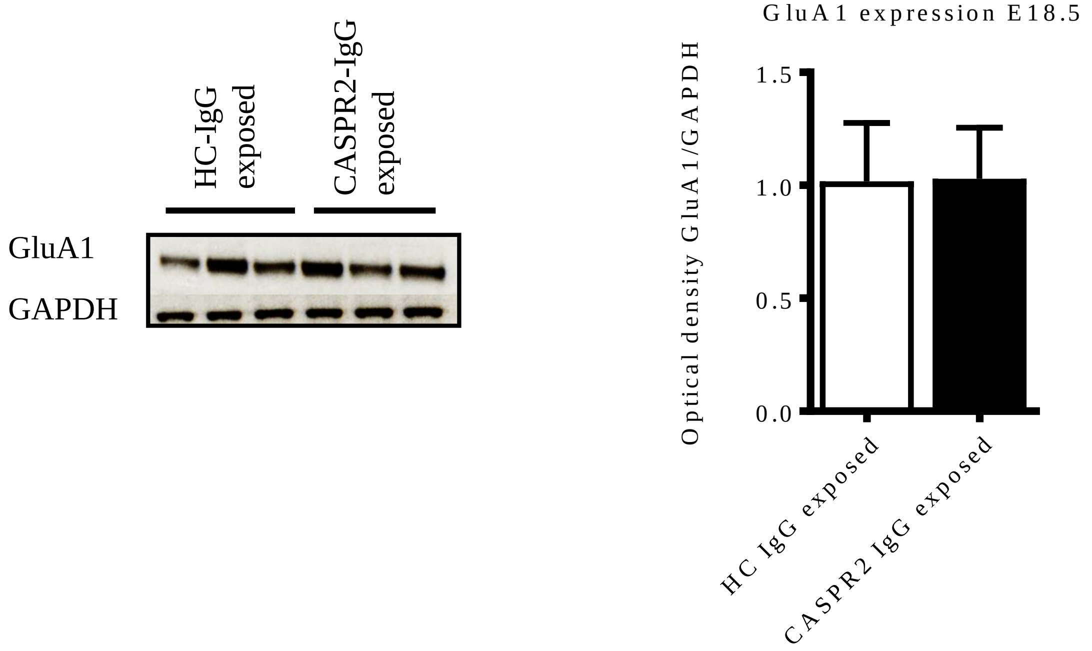

Online Resource 16. Total GluA1 expression in CASPR2-IgG and HC-IgG exposed embryos at E18.5

Data presented as mean ± SEM of two western blot replicates from three mice from each group
